# PrOMet: Accurate and Robust Metal‐Binding Site and Type Prediction

**DOI:** 10.1002/advs.78003

**Published:** 2026-10-11

**Authors:** Gangeun Park, Chaok Seok, Hahnbeom Park

**Affiliations:** ^1^ Department of Chemistry Seoul National University Republic of Korea; ^2^ Galux Inc Seoul Republic of Korea; ^3^ Biomedical Research Division Korea Institute of Science and Technology Seoul Republic of Korea

## Abstract

Metal binding to proteins is crucial for many biological functions, yet the prediction of metal‐binding sites and metal types remains underexplored compared to other biomolecular interactions. Despite advances in deep learning, computational methods have achieved limited success in this area; even the best‐reported approaches reach only moderate localization accuracy on high‐resolution crystal structures, and even worse when applied to medium‐resolution data. We hypothesize that the main challenge lies in the nature of metal binding. While the putative sites span the entire protein surface, actual binding requires the atomic‐level coordination geometry. We present a structure‐based metal binding site and type predictor, PrOMet, in this work, which leverages a Prune‐and‐Optimization strategy built upon two hierarchically connected machine learning architectures. This strategy allows the method to effectively narrow the search space while maintaining robustness to AlphaFold models and medium‐resolution cryo‐EM structures. PrOMet outperforms existing methods, including structure‐based and language model‐based predictors, achieving a PR‐AUC of 0.739 for metal ion localization and an average F1‐score of 0.743 for metal type classification—Zn, Ca, Mg, Mn, Fe, Cu, respectively. PrOMet can be an effective tool for allocating uncertain metal positions and types within protein structures, as well as for metalloenzyme design studies.

## Graphical abstract

PrOMet is a two‐stage deep learning framework for jointly predicting metal‐binding sites and ion identities from protein structures. Surface candidate sites are first pruned, followed by refinement of the most probable sites and metal types. This hierarchical strategy enables robust and accurate metal prediction for cryo‐EM and AlphaFold‐derived structures, consistently outperforming previous methods.

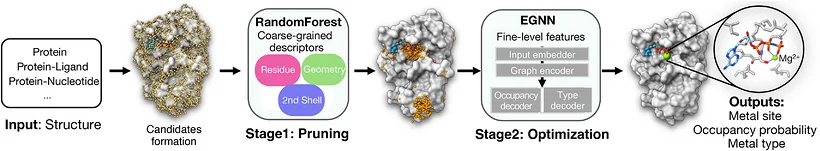

## 1 Introduction

Metal ions play essential roles in biology, including catalysis [1, 2], structural stability [3], transport [4], electron transfer [5], and regulation, and are known to exist in over a third of Protein Data Bank (PDB) structures [6]. Yet, identifying metal binding sites and ion types remains challenging, especially in moderate or low‐resolution maps, where metals are hard to distinguish from solvent or nearby atoms [7, 8, 9]. These challenges highlight the need for accurate computational methods to predict metal‐binding sites from structure alone. Such computational tools should help understand metal‐mediated biological events, but also for optimizing metal‐mediated protein functions, such as *de novo* metalloenzyme design [10, 11, 12] or metal‐dependent activity engineering [13].

There are three groups of research directions regarding the metal site prediction: i) sequence‐only prediction to estimate residue‐level coordination preference [14, 15], ii) structure‐based predictions leveraging 3D protein coordinates based on geometrical heuristics [16] or on deep‐learning feature extraction [17], and finally iii) template‐based methods [18, 19] using known metal‐bound structural information. Each type of approach has its own advantages and limitations. Sequence‐based methods scale well but lack 3‐D context, making precise prediction difficult. Structure‐based approaches have limited applicability to structures with errors or uncertainties. Template‐based approaches can hardly be generalized to unseen proteins. Finally, two common limitations exist regardless of the approach: first, the majority of prediction methods just find sites without the metal type specification, and second, they cannot be utilized for those jointly coordinating with other biomolecules [20]. Given these limitations, a more universal metal prediction method is demanded that works robustly and accurately for broader types of problems.

In this work, we introduce PrOMet (Prune‐and‐Optimization for Metals), a two‐stage structure‐based metal binding site and type prediction framework. The main hypothesis in this work is that a structure‐based method can provide a universal solution once it can address the mixed‐scale nature of the task: scanning the whole protein surface while evaluating fine atomic‐level coordination. In PrOMet, a high‐recall pruning step narrows the global search across the protein surface, and a fine‐grained optimization step enforces coordination geometry and local chemistry to localize sites and assign metal types. PrOMet is, to our knowledge, among the first structure‐based deep‐learning methods that accepts ligand‐bound inputs (small molecules, RNA, or DNA) for predicting both 3‐D location and the metal ion identity.

## 2 Results

### 2.1 Overview of the Work

An overview of the model workflow and architecture is presented in Figure 1. The framework operates in two sequential stages: the Pruning stage followed by the Optimization stage, which are designed for their own distinct purposes. Conceptually speaking, at the first Pruning stage, coarse‐grained geometric filters are applied to remove unlikely patches in a protein surface. At the second Optimization stage, a fine‐grained atomic‐level score is applied to select and rank the most plausible binding locations and types among selected patches. Conceptual overview of each model is provided below; details of input features and model architecture are reported in Methods and Figure S1.

**FIGURE 1:**
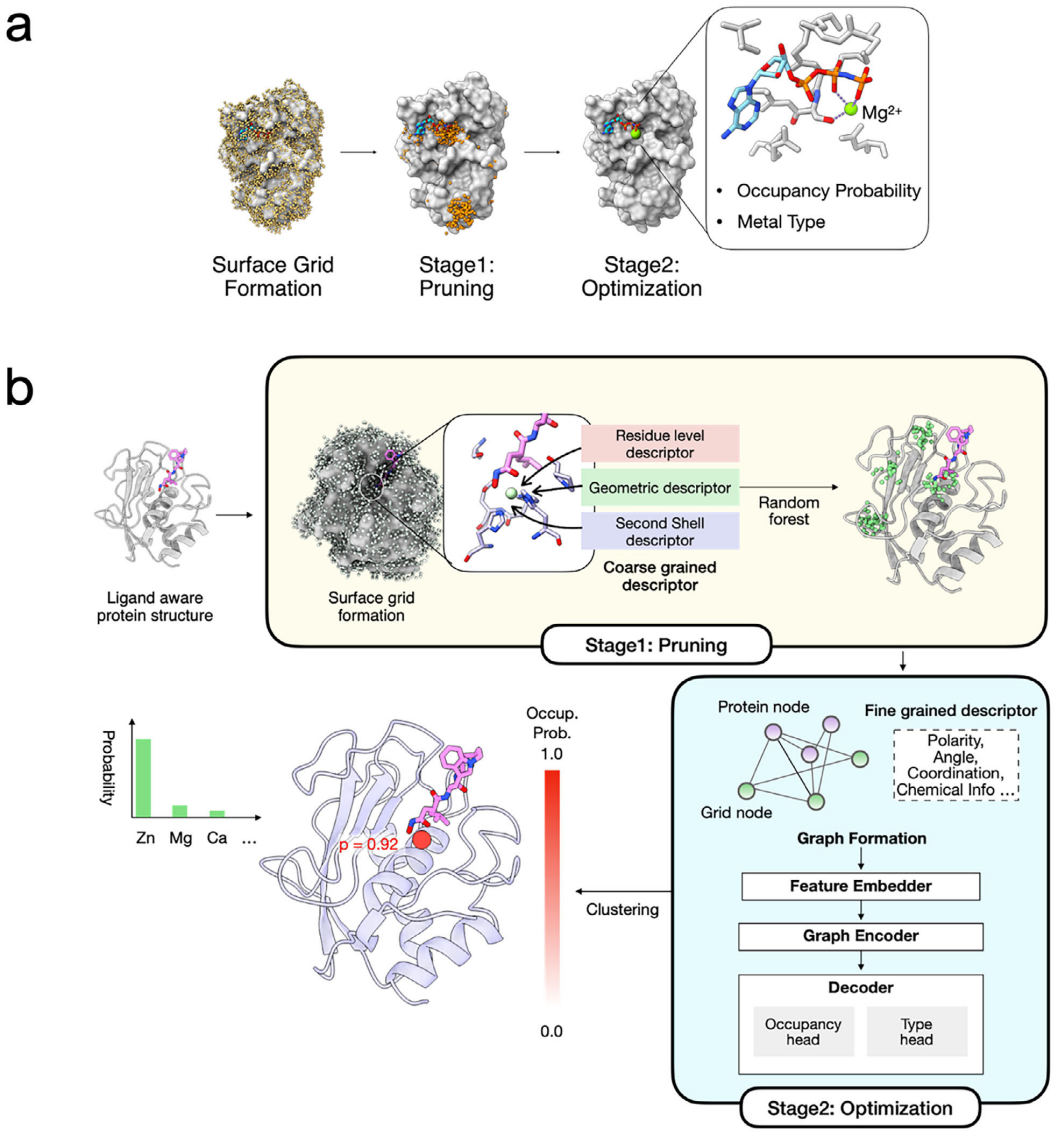
Overview and workflow of PrOMet. (a) Conceptual overview of PrOMet, which predicts metal‐binding locations and metal types on protein structures using a two‐stage framework. (b) The two‐stage workflow is demonstrated: a pruning stage that filters unlikely surface patches using coarse‐grained descriptors and random forest, followed by an optimization stage that builds a protein–grid graph with fine‐grained atomic descriptors to score site occupancy and metal type.

In the first stage, a Random Forest (RF) classifier aims to reduce binding site candidates. The classifier operates on solvent‐accessible grid points on the protein surface, where coarse‐grained features are collected from nearby atoms, such as the number of nearby electron donor atoms and core residues (Asp/Glu/Cys/His), ligand/donor presence, and accessibility. Four core residues are chosen because imidazole N (His), thiolate S (Cys), and carboxylate O (Asp/Glu) are the most common electron donors in protein [21, 22, 23]. We include geometric features that capture pocket backbone shape and steric fit — e.g., distances to the surrounding backbone and carbon framework and steric clash checks with aliphatic/aromatic groups — and the second‐shell context (3.2–5.0 Å), which summarizes non‐coordinating neighbors that stabilize true sites via hydrogen bonding, electrostatics, steric scaffolding, and solvent exclusion [24, 25]. The pruning stage was designed to satisfy two requirements simultaneously: retaining true metal‐binding regions with minimal loss and reducing the candidate grid set to a size manageable for the downstream optimization stage. Accordingly, the filtering criterion was chosen to maximize metal‐site coverage while strongly reducing the search space. A metal‐binding site was regarded as covered when at least one retained grid point was located within 2 Å of the annotated metal position. On the independent test set, this pruning step achieved 98.8% metal‐site coverage while retaining only 2.76% of the initial search space (i.e. grid points), corresponding to the removal of 97.24% of the original candidates. The grid‐level false‐to‐true ratio was reduced from 1224:1 before pruning to 37:1 after pruning.

In the subsequent optimization stage, the likelihood values of these candidate grid points with probability ≥ 0.5 are clustered and re‐ranked, along with the prediction of the binding metal type. Here we switch to fine‐grained descriptors that resolve local coordination geometry—e.g., donor–grid distances, an atom's chemical type, and an angle between each atom's polarity vector and the grid direction. The binding site candidates were allocated to the probability‐weighted cluster centers, and metal types are predicted through an independent head. The scoring network was implemented using the Equivariant Graph Neural Network (EGNN) [23, 26].

Both the RF classifier and EGNN scorer were trained on 16,196 PDB entries encompassing protein‐metal complexes determined either by X‐ray crystallography or cryo‐EM. In contrast to previous works in which only high‐resolution structures were taken, our dataset spans the broad resolution spectrum reported in the PDB (Figure S2) and all ligands and nucleic acids except waters were retained. This choice was intended for two reasons: first, we sought a network generally applicable to medium‐resolution or computationally modeled structures and in the presence of biologically co‐bound molecules, and second, to extend the dataset as much as possible. To reduce non‐biological metals [7], we applied filtering based on the number, types, and proximity of coordinating atoms. For a fair evaluation, we enforced chain‐level non‐overlap with a 30% sequence‐identity clustering between training and held‐out test sets, and constructed independent benchmark subsets (crystal, cryo‐EM, AF3) from this split (Table S1).

### 2.2 PrOMet Accurately Localizes Metal‐Binding Sites in Experimentally Determined Protein Structures

We first benchmarked the metal binding site prediction on experimental protein structures, a metal‐type agnostic task. Three approaches were selected for comparison, representing different types of approaches: Metal3D [17, 27] for a structure‐based deep learning method, BioMetAll [16] for a heuristic and geometry–driven method, and M‐Ionic for a sequence‐based method [7, 28]. For a fair comparison across methods, we evaluated all models on our holdout test set, ensuring that no test protein shared more than 30% sequence identity with any protein in our training set (see Methods for details).

In Figure 2a, we compared site‐level precision–recall (PR) curves. A prediction is considered correct if the predicted positional deviation from the answer is within two cutoffs: 2 and 5 Å for precise and tolerable analyses. At both thresholds, PrOMet achieved the highest PR‐AUC (area under curve) compared to all the other methods. Using a 2 Å cutoff and threshold 0.5, PrOMet achieved a recall of 80.0% and a precision of 65.0%, which is superior to others (the second best Metal3D, recall/precision = 75.8/37.2%). To compare against a residue‐level predictor M‐Ionic, we evaluated the precision and recall of coordinating residue prediction defined as <3 Å to the metal (Figure 2b). Again, in this metric, our model achieved a higher PR‐AUC (0.791 vs 0.617).

**FIGURE 2:**
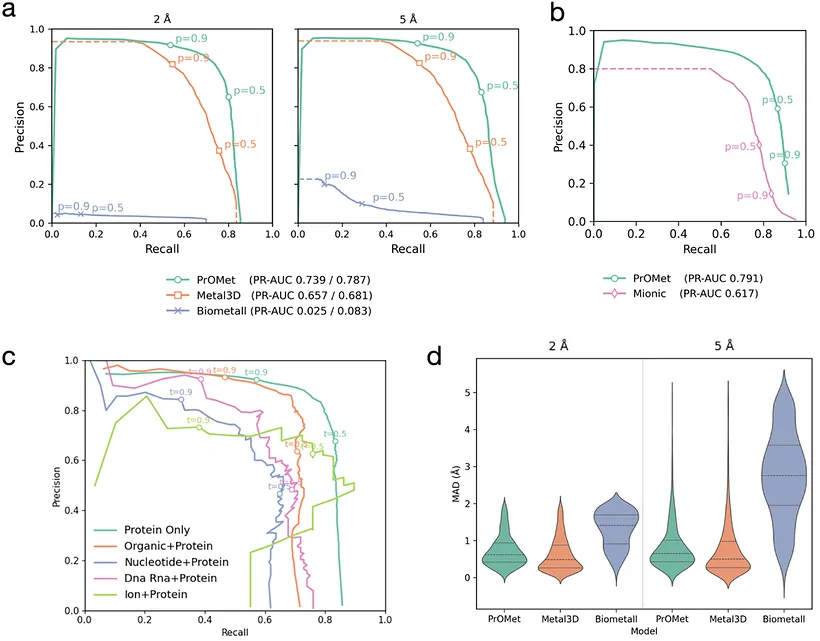
Performance comparison of metal‐binding site prediction methods in precision–recall (PR) curves. (a) Metal‐site–level predictions with evaluation distance thresholds of 2 Å (left) and 5 Å (right). (b) Residue‐level predictions. (c) Per‐ligand type prediction of PrOMet. (d) Distribution of metal site positional errors among correctly predicted sites at 2 Å (left) and 5 Å (right).

Decomposing the prediction accuracy by whether coordination involves molecules other than proteins, a unique utility of PrOMet over other published works, PrOMet broadly works for various molecular interfaces (Figure 2c). A slight drop in non‐protein interfaces might be due to less training data; however, to our knowledge, unlike other published works, it is encouraging to first demonstrate that metal sites can be reasonably predicted to broader types of biomolecular interfaces, including protein‐ligand, protein‐nucleic acids, and on. As an illustrative example, we analyzed PrOMet predictions on Mg^2^^+^ binding sites of RuvAB AAA^+^ ATPase complexes (PDB 7PBR/7PBP) [29] (Figure S3). This target is a non‐trivial CASP15 ligand target only a few groups predicted correctly, in which Mg^2^^+^ occupancy depends on the nucleotide state. PrOMet correctly localized Mg^2^^+^ only to ATPγS‐bound subunits and predicted its absence at ADP‐bound pockets, reproducing the experimental occupancy pattern around the ring. Performance was unchanged in 7PBP despite the presence of RuvA, indicating that the method conditions on the local context rather than merely memorizing motifs.

The binding site prediction accuracy by PrOMet generally worked for all metal types. According to the analyses decomposed to metal types Zn, Ca, Mg, Mn, Fe, and Cu (Figure S4), PrOMet achieved a good balance between precision and recall values across the 6 most common metals. Interestingly, although Metal3D was trained solely on Zn‐binding data, PrOMet still achieved comparable performance for Zn‐binding site prediction (best F1 score 0.83 vs 0.82) (Figures S4 and S5).

Finally, we evaluated the structural accuracy of predicted metal‐binding site coordinates (Figure 2d). This analysis measures how exactly the coordinates can be estimated when the binding site was predicted correctly. At the 2 Å cutoff, PrOMet and Metal3D showed comparable sub‐Ångstrom accuracy, with median errors of 0.620 Å and 0.484 Å, respectively. Although Metal3D achieved a slightly lower median error, PrOMet correctly localized substantially more sites (2,340 vs. 2,017). A similar trend was observed at the 5 Å cutoff, where PrOMet maintained coordinate accuracy comparable to Metal3D while matching more binding sites (2,444 vs. 2,072; Table S2).

### 2.3 PrOMet Reliably Distinguishes Between Different Types of Bound Metal Ions

Next, models were benchmarked in their ability to jointly predict the binding site and metal type. The comparison could be made only between PrOMet and M‐Ionic; BioMetAll and Metal3D provide sites only. Models were again benchmarked on our test set ensuring that no test protein shared more than 30% sequence identity with any protein in our training set. A prediction is regarded as correct if the site prediction is within 5 Å and the highest probability metal type matches the actual type. For the residue‐level predictor M‐Ionic, residue‐level signals were first converted to sites by averaging the centers of coordinating atoms in predicted residues, then by assigning a metal type with the highest probability. Note that M‐Ionic was not designed to predict binding‐site coordinates; consequently, coordinate predictions derived from its predicted contact residues are expected to exhibit substantially lower accuracy. In Figure 3a, per‐metal type benchmark results on the test set are reported. PrOMet attained higher precision compared to M‐Ionic, resulting in a higher F1‐score for every metal class in our evaluation. Note that Co and Ni had very few data and were ignored from the analysis. Gains were more pronounced for well‐represented ions: Zn improves from 0.343 (M‐Ionic) to 0.859, and Ca from 0.193 to 0.801.

**FIGURE 3:**
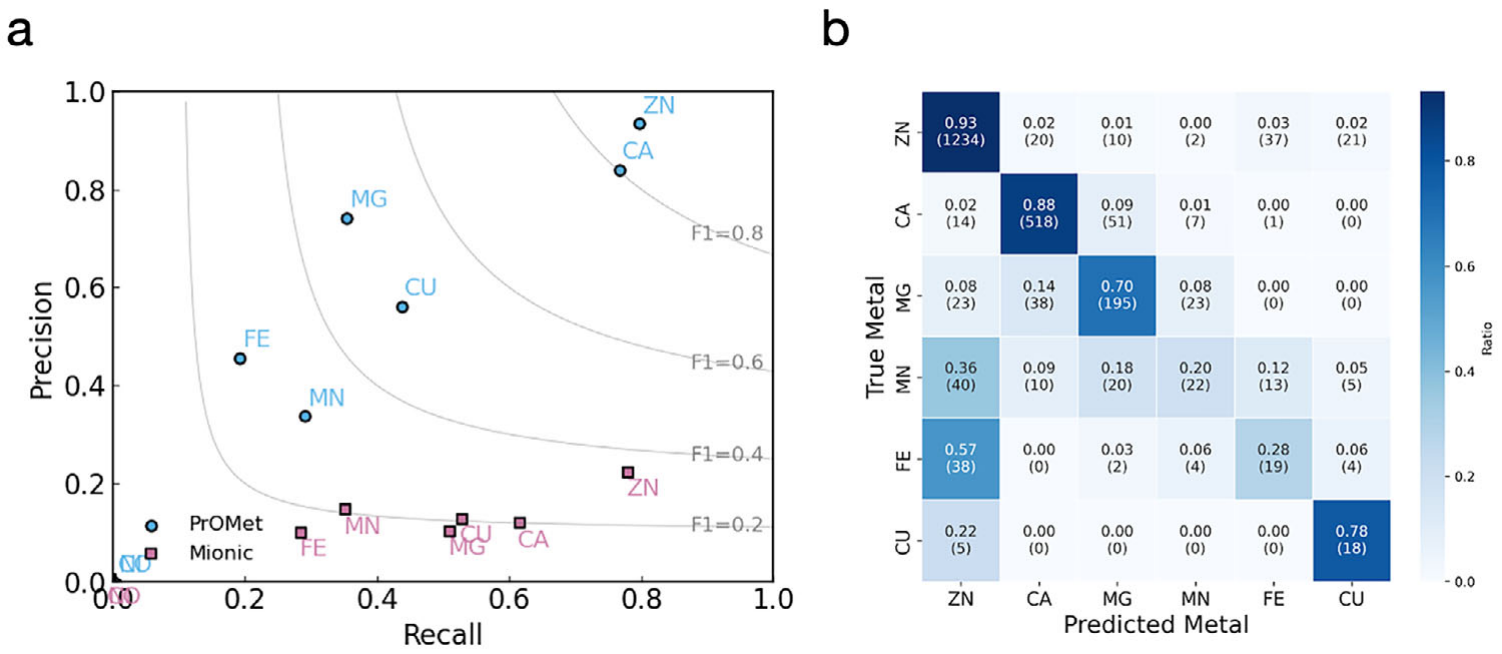
Joint evaluation of binding‐site and metal‐type prediction.(a) Per‐metal precision–recall summary for PrOMet (circles) and M‐Ionic (squares) on the test set. A prediction is considered correct when the predicted site is within 5 Å of the annotated metal and the highest‐probability metal type matches the true type. PrOMet achieves consistently higher precision and F1‐score than M‐Ionic across all evaluated ions; Co and Ni are omitted due to insufficient data. (b) Confusion matrix of PrOMet metal‐type predictions, showing for each true ion the fraction of sites assigned to each predicted ion. Counts are shown inside parentheses below fraction values. Most predictions lie on the diagonal, while misclassifications tend to occur between chemically similar ions, such as Ca–Mg ions [30].

To better understand PrOMet's behavior beyond statistics, we examined qualitative patterns found in successful and failed predictions. First, certain prediction errors were still chemically consistent: a confusion analysis (Figure 3b) shows predictions were often swapped more systematically than randomly between chemically similar ones, such as alkaline‐earth cations (Ca and Mg) [31, 32]. This pattern may also be influenced by the dataset composition. Because the training set includes experimental examples in which very similar proteins are observed with different bound metals, the model is exposed to realistic metal substitutions and may therefore learn chemically plausible confusions between similar ions. Second, according to the confusion matrix, Zn was over‐represented and many Mn or Fe sites were predicted to be Zn‐binding instead (low precision‐recall for these metal types), reflecting dataset imbalance. Third, despite the imbalance, a relatively good performance was observed for the sparse metal type Cu, which is attributed to unique local coordination geometry (e.g., His/Cys‐enriched coordination and characteristic geometric distortions) [22, 33].

### 2.4 PrOMet Maintains Robust Performance on Non‐Crystallographic Structures

One of the well‐known weaknesses of a structure‐based approach over a sequence‐ or a template‐based approach is its undesired sensitivity to structural noise. To investigate the tolerance of methods against structural uncertainty, we benchmarked methods in two ways. First, by conditioning on structure determination methods (X‐ray crystallography versus cryo‐EM), all three methods – PrOMet, Metal3D, and BioMetAll – show performance decreases on cryo‐EM relative to crystal structures (Figure 4a) when used as structural inputs (resolutions of inputs used in this study reported in Figure S6), yet the amount of decrease was comparable across methods.

**FIGURE 4:**
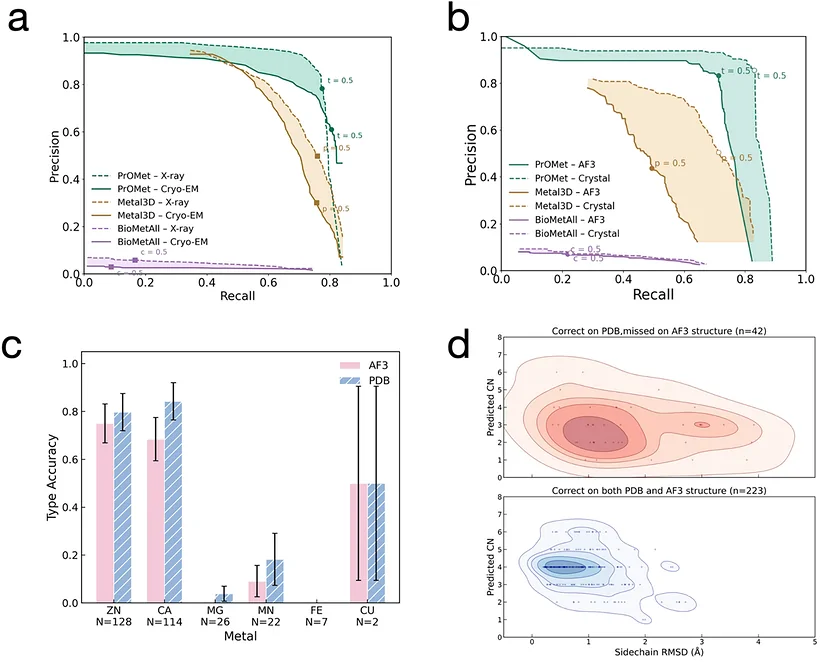
Robustness of metal‐binding site prediction to structural input types. (a‐b) The performance difference between two structural input types are evaluated for PrOMet (green), Metal3D (yellow), and BioMetAll(purple). The performance gaps are highlighted by colored areas. (a) Evaluation by conditioning on structure determination methods: X‐ray crystallography (dotted) versus cryo‐EM (solid). (b) Evaluation on AlphaFold3 (AF3) models (solid) versus experimental crystal structures (dotted). (c) Per‐metal type accuracies for AF3 versus experimental structures, evaluated for 6 major ions (Zn, Ca, Mg, Mn, Fe, Cu). (d) Relationship between input model type and two contributing factors: pocket sidechain RMSD (x‐axis) and predicted coordination numbers in model structure(y‐axis). Probability distribution contours are shown for two cases: Red) incorrect with the AlphaFold model but correct with the crystal structure; Blue) correct for both models.

Because predicted structures are increasingly used, we next asked whether PrOMet maintains accuracy on AlphaFold (AF) model inputs [34]. Before running predictions, we first analyzed the structural accuracy around metal binding sites (residues around ≤ 5 Å the metal) in AFDB models (for sufficient statistics) [35], and found backbone and sidechain structures were quite accurate with 90% having small RMSD values of ≤ 0.5 Å and ≤ 3 Å, respectively (Figure S7). When metals were copied into the aligned structural models, donor–metal atomic distances were reasonable but generally showed broader variance with respect to experimental structures (Figure S8).

We then benchmarked prediction methods on the AF3 model structures [36] (modeled without metal inputs) for an accurately modeled subset. The subset contained precise models of 200 targets in the test set, in which metals bind to a single chain only, having global backbone RMSD ≤ 5 Å and local side‐chain RMSD ≤ 3 Å at the binding pocket. Evaluating site and type predictions, PrOMet again showed only a small degradation relative to their crystal structures, whereas Metal3D dropped more sharply (Figure 4b). The metal type ranking was consistent between experimental and AF3 structures (Figure S9).

To examine how metal‐type predictions change when using model structures, we evaluated the joint site–type accuracy on AF3‐generated models, requiring both the binding site and metal type to be correct. Performance on AF3 models showed only a modest decrease compared with the corresponding crystal structures (Figure 4c). Consistently, confusion matrices computed using experimental versus AF3 structures (Figure S9) exhibited highly similar patterns. These results indicate that, in scenarios where AF3 provides reasonable binding pocket structures, PrOMet remains robust despite potential structural inaccuracy. Analyzing the cases when the prediction was poor for AF3 models, failures were dominant when both coordinating side‐chains were modeled poorly (RMSD > 2 Å) and the coordination number was too low [8] (less than 3, Figure 4d). Consistent with this, cases remained correct when several donor atoms were still present even with some local distortions.

### 2.5 Benchmarking AlphaFold3 Metal Modeling Highlights the Need for Dedicated Metal‐site Prediction Tools

AlphaFold3 and related modeling tools [27, 37] now provide a utility to predict protein‐metal complexes. Here we benchmark their “site and type prediction” ability, rather than their original “structure‐prediction” ability, to investigate the complementary role of dedicated methods such as PrOMet given the presence of such powerful tools. In practice, these methods are used in different contexts: AlphaFold3 (AF3) places metals given the user specification of the metal count and types, while PrOMet requires no metal‐related specification but a protein structure as input, outputting both metal‐binding locations and types.

We tested two tasks here: i) whether a method can predict non‐binding examples correctly as non‐binding, and ii) whether a method can predict the correct metal type at a known binding site. For the first test, we collected a negative set of 316 proteins with no reported metals and prompted AF3 with one Zn. For the second task, we collected a positive set of 82 monometal proteins. Two sub‐tasks were made with AF3: in the “All‐metal” task, all 7 ions (Cu, Fe, Mg, Zn, Ca, Mn, K) were prompted simultaneously, and the “Per‐metal” task, one metal was prompted at a time and repeated 7 times. To fairly benchmark two methods, these test proteins are collected from previous work [38], further applying sequence identity filters to the PrOMet training data by <40%.

For the first non‐binding discrimination task, AF3 tended to place a metal with high confidence. Of 316 negative proteins tested, 45 cases had a pLDDT ≥ 80 and 10 had a pLDDT ≥ 90, indicating that placements alone do not separate negatives from positives (Figure S10). This implies AF3 can often overestimate metal‐binding, which is likely originating from the fact that no such supervision was made in its training. Applying PrOMet to the same dataset, the model predicted metal‐binding sites in 26 cases (8.2%) at a probability threshold of 0.8, indicating that false positives can occur in PrOMet results as well (Figure S11). Visual inspection of these cases (Figure S12) revealed that the majority of PrOMet failures were associated with incorrect guesses on histidine tautomer states or corresponded to sites that still appeared plausibly capable of metal binding. In contrast, many sites predicted by AlphaFold3 were generally less likely to bind metals and potentially reflecting effects of data memorization (Figures S13 and S14).

For the second type prediction task, AF3 was able to predict the type and site correctly for 23% of cases (Figure 5a). All‐metal and Per‐metal settings achieved 61% site‐correct cases. However, the Per‐metal strategy showed better type discrimination and ranking of the correct site, increasing the fraction of site‐and‐type‐correct predictions from 29% to 51% when all predictions were counted, and improving the top‐1 pLDDT site‐correct rate from 35% to 49%. Despite this, the final top‐1 site‐and‐type‐correct rate remained 23% in both settings. This is inferior to ProMet, which achieved top‐1 site‐and‐type accuracy of 45% in the same dataset, while requiring significantly more computing resources (inference runtime using RTX A5000 on 8y4u) was **1.53 s** for ProMet (featurization + inference), **126.0 s** for AF3 all‐metal, and **139.6 s × 7** for AF3 per‐metal (seven independent runs with the same condition).

**FIGURE 5:**
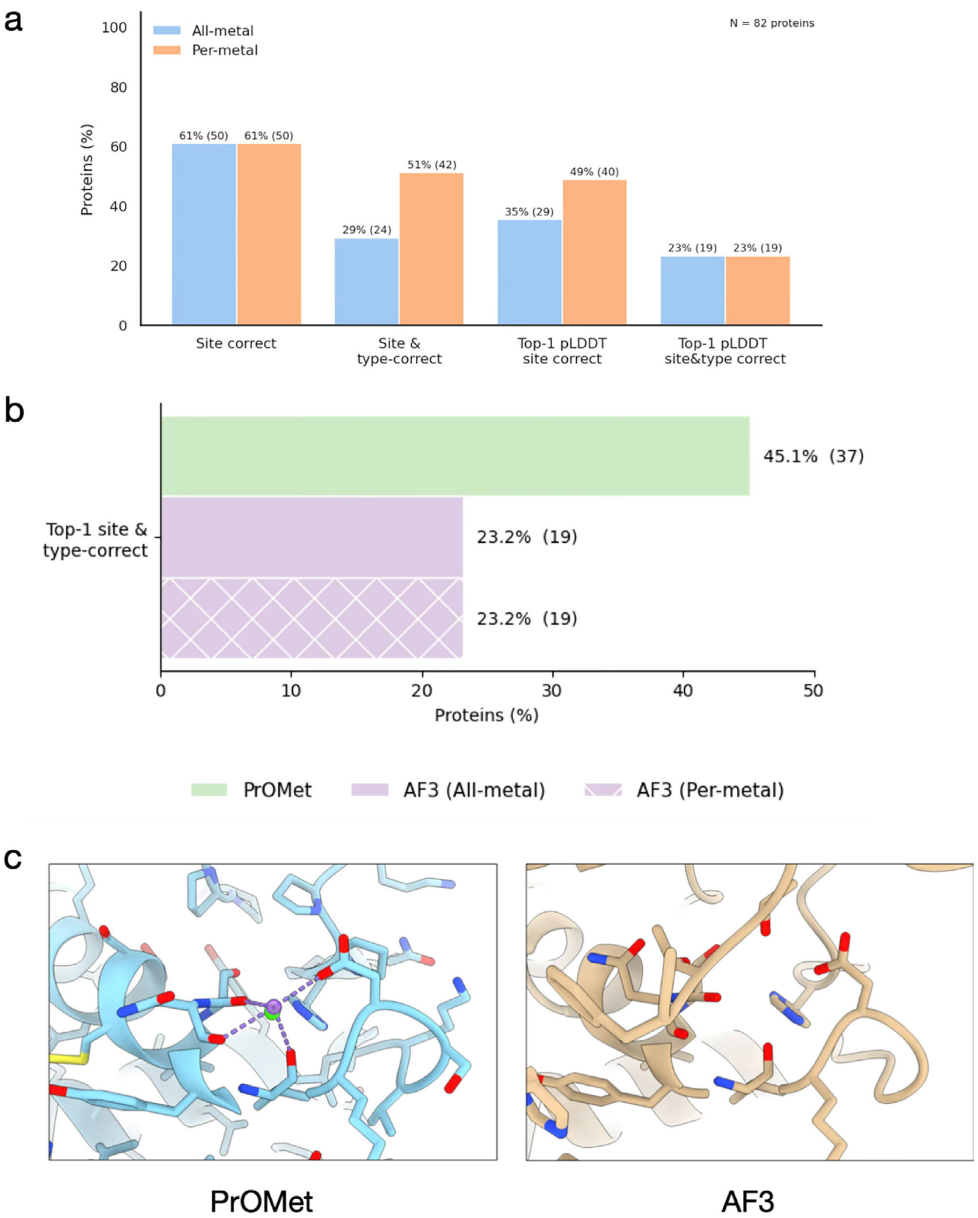
Performance of AlphaFold3 (AF3) on predicting metal binding site and type. 82 monometal proteins with known metals are benchmarked. (a) AF3 results under two prompting schemes are shown: “All‐metal” (Cu, Fe, Mg, Zn, Ca, Mn, K simultaneously) and “Per‐metal” (one ion at a time). Bars show the fraction of proteins (with counts) satisfying each criterion: at least one correct site, at least one site and type correct, top‐1 pLDDT site correct, top‐1 site and type correct. All‐metal prompts and Per‐metal prompts yield same counts of site hits, whereas Per‐metal prompts give a higher proportion of type‐correct predictions, indicating that pLDDT alone is insufficient for metal‐type discrimination. (b) Comparison of ProMet and AlphaFold3 (AF3) for predicting metal‐binding sites and metal types. Horizontal bars report the fraction of proteins (percentage with counts in parentheses) for which each method achieves at least one prediction with both correct site and metal identity (*Site & type‐correct*). AF3 is evaluated under two prompting schemes: All‐metal (multiple candidate ions provided simultaneously) and Per‐metal (one ion queried at a time). ProMet is applied to AF3‐predicted structures and shows higher success rates than AF3 (All‐metal and Per‐metal) under both criteria, highlighting the value of a dedicated metal‐site predictor as a complementary tool to structure prediction methods. (c) Example of a K^+^ site missed by AF3 but correctly identified by PrOMet. In TsCWBD–GH24 (PDB: 8B2S), AF3 failed to place any ion in the SH3‐like cell‐wall‐binding pocket under either prompting scheme, whereas PrOMet accurately predicted both the site and K^+^ type.

As an illustrative example, the fungal GH24 muramidase TsCWBD–GH24 (PDB 8B2S) contains a K^+^ ion modeled in a pocket of the SH3‐like cell‐wall‐binding domain in the X‐ray structure. AF3 failed to place any metal ion in this site under either the all‐metal or per‐metal prompting schemes (Figure 5c). In contrast, PrOMet correctly predicted the site and type when applied to the AF3 backbone. This difference is likely due to the specific coordination chemistry involved; having three out of five atoms as backbone carbonyls should be an underrepresented example, and hence informatics‐based approach is more likely to fail. Overall, our comparative study highlights the need for a dedicated metal‐site predictor as a complementary tool to structure prediction tools like AF3.

### 2.6 Ablation Analysis Reveals Contributions of Two‐Stage Design, Directional Features, and Dataset Curation

Through further ablation studies, we tested to what extent PrOMet benefits from a two‐stage protocol. When the fine‐grained EGNN scorer was tested without the prior pruning stage, the PR‐AUC value significantly dropped from 0.739 to 0.386 (the Model section in Table 1, details in Figure S15). The main reason was a large increase in the number of false positives, which dramatically reduced precision and, in turn, lowered the F1‐score. We thought this could be an artifact from the fact that EGNN scorer was trained on a pre‐filtered dataset. To see the best performance without such an artifact, we retrained the EGNN scorer on a non‐filtered set (No RF retrained in the table), but the performance was still poor. These results reveal two‐level filters were necessary not only for efficiency but also for prediction accuracy. This ablation study strengthens our major hypothesis underlying this work, that the dominating factor to the successful prediction by PrOMet is its two‐stage architecture design.

**TABLE 1:** Ablation analysis of PrOMet training data, model, and input features.

Factor	Setting	Dataset	F1‐score	Precision	Recall	PR‐AUC
Model	Base (PrOMet)	General PDB	**0.721**	**0.656**	**0.800**	**0.739**
	No RF	General PDB	0.142	0.078	0.814	0.386
	No RF (re‐trained)	General PDB	0.205	0.435	0.134	0.205
Input feature	No polarity‐vector angle	General PDB	0.653	0.572	0.760	0.609
Dataset	Base (PrOMet)	CryoEM	**0.653**	0.543	**0.818**	0.774
	High‐res only	CryoEM	0.627	0.547	0.736	0.646
	No label filtering	CryoEM	0.623	**0.629**	0.617	0.664
	Base (PrOMet)	X‐ray	**0.779**	0.783	**0.775**	0.753
	High‐res only	X‐ray	0.747	0.807	0.695	0.716
	No label filtering	X‐ray	0.606	**0.837**	0.475	0.528

We then investigated the impact of input features. We ran an ablation study on various input features in EGNN scorer, and found out that the directional feature was most critical. This feature includes the polarity vector and the grid orientation angle (θ). We observed a decrease in precision removing them, leading to lower F1 and PR‐AUC (Table 1 and Figure S16). Other features tested were not very critical, removing them resulted in only small differences. This pattern suggests that the directional feature provides an inductive bias that effectively constrains the search space for metal‐binding sites. From a physical perspective, this feature encodes the orientations of lone‐pair electrons from their nuclei, providing critical information for metal coordination. Such orientation‐dependent information cannot be captured by distance‐only metrics, highlighting the advantage of equivariant graph architectures, such as EGNN, over conventional graph neural networks.

In addition to the dominant contribution of the two‐stage architecture, dataset composition and label curation further improved robustness to structurally noisy inputs. First, training data include both crystal and cryo‐EM structures spanning high to medium/low resolutions, repeatedly exposing the model to coordinate noise and partial distortions. This distributional exposure was likely preventing overfitting to perfect geometry and guided networks to learn tolerances to moderate structural noises. Second, we strictly selected biological sites as training targets via chemically motivated heuristics (number/types/proximity of donor atoms, details in Methods), filtering out crystallographic artifacts and non‐biological binding. These results suggest that the model consistently learned chemical constraints, such as coordination number, donor preferences, distance/angle bounds, and naturally rejected chemically implausible arrangements even when input coordinates were noisy. Consistent with this mechanism, training ablations (the Dataset section in Table 1) show that training only on high‐resolution structures mainly reduces recall and depresses overall F1‐score. No label filtering also showed a decrease in F1‐score; it improved precision at the cost of decreased recall, highlighting that both ingredients were necessary for robustness.

Finally, we performed a dataset and feature ablation analysis for each metal type to address two questions: (i) what accounts for the overall performance gap between PrOMet and Metal3D, and (ii) which features are most critical for accurately predicting each metal type. Training the model exclusively on Zn, as in Metal3D, resulted in a substantial loss of accuracy for alkaline‐earth metals (drop in PR‐AUC by 0.39 for Ca and 0.27 for Mg), while maintaining reasonable performance for the rest (Zn, Mn, Fe, and Cu). This finding largely explains the overall performance difference between PrOMet and Metal3D (Figures S17 and S18). Furthermore, removing polarity‐related features caused larger decreases in recall for Mn and Cu than for Ca and Mg (Figure S18). For Cu, this dependence is consistent with its anisotropic and frequently distorted coordination environments. For Mn, the polarity feature may instead help distinguish the spatial organization of its predominantly oxygen‐rich coordination sites from superficially similar Mg‐ and Ca‐binding environments. Architecture replacement with GAT or GCN also reduced performance, although the magnitude of this effect varied across metal classes.

#### 2.6.1 Model Choice for the Two‐Stage Framework

The two stages of PrOMet address distinct subproblems and therefore use different model classes. In the pruning stage, the input consists of hand‐crafted tabular descriptors that summarize the local chemical environment, steric accessibility, and second‐shell context around each candidate grid point. For this type of structured data, tree‐based ensemble ML methods provide a strong and efficient solution. We adopted a Balanced Random Forest because the main requirement of the pruning stage is to retain true metal‐binding regions with very high coverage, while substantially reducing the candidate search space for the downstream optimization stage.

As additional comparisons, we evaluated both LightGBM and a simple deep‐learning baseline, a 3‐layer MLP, on the same task (Table S3). On the independent test set at a threshold of 0.5, Random Forest and LightGBM showed comparable performance: metal‐site coverage was 98.8% and 98.7%, respectively; the retained grid ratio after filtering was 2.76% and 2.52%; and the false‐grid to true‐grid ratio after filtering was 37.0 and 33.9. By contrast, the MLP was much more aggressive in filtering, retaining only 0.06% of grids and reducing the false‐grid to true‐grid ratio to 0.615, but this came at the cost of substantially lower metal‐site coverage (84.5%). These results indicate that although the MLP can generate a cleaner candidate set, it removes too many true metal‐binding regions to be suitable for the pruning stage. Overall, the results support tree‐based models as more appropriate for this high‐recall filtering problem, with Random Forest and LightGBM offering similar recall–filtering trade‐offs. We chose Random Forest as a simple and robust default. Additional threshold‐sensitivity analyses showed that the downstream performance remained relatively stable for pruning thresholds between 0.4 and 0.7, while the highest F1‐score was obtained at 0.7. We nevertheless used 0.5 as the default threshold, as it provides a balanced operating point, and thresholds in the range of 0.4–0.7 may be selected depending on the desired precision–recall trade‐off for a given application (Figure S19).

By contrast, the optimization stage requires explicit reasoning over local 3D coordination geometry, atom identities, and directional relationships between protein atoms and candidate grid points. We therefore employed an Equivariant Graph Neural Network (EGNN), which naturally accommodates geometric learning on 3D graphs while preserving equivariance to rigid‐body transformations. To support this design choice, we compared EGNN with two additional graph‐based baselines, GAT and GCN, in the same optimization‐stage setting. EGNN achieved the highest performance, with a PR‐AUC of 0.740, followed by GAT (0.579) and GCN (0.546) (Figure S20). This ordering suggests that explicit modeling of geometric relationships is important for the task. The stronger performance of GAT relative to GCN further indicates that adaptive weighting of neighboring nodes is beneficial, but remains insufficient to match a model that directly incorporates local geometric structure. Taken together, these comparisons support EGNN as a practical and well‐motivated choice for the optimization stage.

## 3 Discussion

### 3.1 Comparison to Existing Methods

Although BioMetAll, Metal3D, MIB2, and PrOMet are all structure‐based approaches, they rely on different assumptions and are therefore suited to distinct predictive settings. BioMetAll identifies geometrically favorable coordination motifs using backbone‐only descriptors and is thus most effective for well‐defined canonical motifs, rather than diverse all‐atom coordination environments. Metal3D serves as a deep learning baseline for structure‐based metal‐site localization, particularly in high‐resolution settings; however, its generalizability is limited by training restricted to Zn‐binding crystal structures and by predicting sites alone without metal type specification. MIB2 is a template‐based method that depends on the availability of related protein structures. In contrast, PrOMet is designed for broader applicability across multiple metal species, heterogeneous coordination environments, and inputs with structural uncertainty. Overall, the observed performance gap between PrOMet and existing methods likely reflects not only differences in model accuracy, but also differences in design goals, training scope, and intended use cases.

Compared with Metal3D and motif‐matching approaches such as BioMetAll and MIB2, PrOMet demonstrates particularly strong performance for alkaline‐earth metals (Ca and Mg), whose coordination environments are generally more oxygen‐rich and structurally diverse. These characteristics make Ca/Mg binding sites more challenging to identify using motif‐matching approaches. Among deep learning–based methods, PrOMet and M‐Ionic (based on its reported results) achieve comparable accuracy to the other metal types while substantially outperforming Metal3D on Ca and Mg, a difference that is largely attributable to whether these metals were included in the training data (Figure S18). We also investigated whether other factors, such as directional features and architectural choices, influence Ca/Mg prediction performance, but their effects were small (Figure S18). These results highlight the ability of deep learning–based methods to generalize across diverse coordination chemistries and accurately predict binding sites for both alkaline‐earth and non‐alkaline metals.

These differences highlight the relative strengths and use cases of each method. PrOMet offers broad applicability and is particularly advantageous when (i) protein structures are modeled or expected to be less accurate, (ii) predictions are required in heterogeneous molecular contexts, such as protein–ligand or protein–nucleic acid complexes, or (iii) joint prediction of metal identity and position is needed. In contrast, the other tools are more specialized in scope: Metal3D is well suited for predicting Zn or transition metal binding sites in high‐resolution structures, whereas BioMetAll and MIB2 are most effective for identifying metal‐binding sites associated with well‐characterized motifs.

### 3.2 The Effect of Class Imbalance Across Metal Types

We observe the imbalance of the dataset for different metal binding proteins may influence the prediction model, as shown in the high performance and over‐preference to Zn compared to the rest (Figure 3a,b). To assess whether metal‐type reweighting scheme changes this behavior, we retrained the model without inverse‐frequency metal weighting in the type‐classification losses, which was originally introduced to alleviate preference to highly represented types. Removing the weighting caused only minor changes in aggregate performance (overall precision/recall/F1: 0.847/0.661/0.743 with weighting vs. 0.851/0.665/0.746 without weighting; class‐balanced F1: 0.401 vs. 0.398, PR‐curve reported in Figure S21), indicating that it is not the relative weight but the absolute diversity of the dataset that determines per‐type predictive power. This suggests it is crucial for minority metals to expand the available training data in future works.

### 3.3 Concluding Remark

This study introduces PrOMet, a deep‐learning model that identifies metal‐binding sites at atomic resolution in protein structures while simultaneously predicting the bound metal species. PrOMet demonstrates consistently strong performance across experimental structures of varying resolutions (X‐ray and cryo‐EM) as well as predicted models (AF3), outperforming previous methods in both metal‐site detection and metal‐type classification. The model further generalizes to a broader range of interfaces—including protein‐only, protein–DNA, protein–RNA, and protein–ligand interactions—representing, to our knowledge, the first systematic exploration of this scope. Ablation studies indicate that this versatility and robustness stem from the model's Prune‐and‐Optimization strategy, which enables high recall without sacrificing precision.

The work can be improved in several directions. First, due to dataset imbalance, minority metal classes (e.g., Mn, Co, K) were underrepresented, resulting in relatively lower performance and less reliable uncertainty estimates for those types. Second, because the current method takes static structures as input, it does not directly capture the temporal and multi‐state rearrangements associated with metal binding (e.g., side‐chain reorientation, conformational change, and long‐range allostery). To address these limitations, promising directions include (i) building large‐scale, standardized benchmarks that better cover minority metal species and non‐protein coordination environments, and (ii) integrating multi‐level ensembles or dynamics (equilibrium ensembles, MD/sampled structures).

To summarize, PrOMet is an accurate and robust tool for joint prediction of metal locations and types. We expect to see its strong potential to drive practical applications in future works, such as correcting uncertain metal placements in deposited experimental structures or designing metalloenzymes.

## 4 Methods

### 4.1 Datasets

#### 4.1.1 BioLiP‐Derived Metal‐Binding Dataset

To build a dataset for biologically relevant metal‐binding site prediction, we used protein–metal complexes from the BioLiP database [38, 39] (retrieved at 2025.02.19). We included proteins containing Mg, Zn, Mn, Ca, Fe, Ni, Co, Cu, or K. Initially, 12 candidate metals with more than 500 associated PDB entries (Mg, Zn, Ca, Fe, Na, Mn, K, Ni, Cu, Co, Cd, Hg) were initially considered [40], but excluded toxic metals (Cd and Hg) and sodium (Na) to avoid buffer‐related artifacts [41, 42, 43, 44]. For each entry, all ligands present in the PDB structure were retained except water molecules.

#### 4.1.2 Biological‐Site Filtering

To distinguish biological from non‐biological metal‐binding sites, an annotated dataset was analyzed from prior work that labels sites as biological or non‐biological [45]. We examined the distribution of coordinating atoms (N, O, S, Se) within 3 Å for both classes and found the greatest separation when at least three coordinating atoms were present (Figure S22). Using that annotated dataset for the threshold validation, the method correctly identified 1,617/1,921 biological sites and 1,770/2,823 non‐biological sites. Based on this analysis, we adopted a ≥ 3 coordinating‐atom threshold to filter sites in our dataset, reducing crystallographic artifacts and focusing on biologically relevant patterns (Figure S22).

#### 4.1.3 Clarification on Proteins Annotated With Different Metals

In deposited experimental structures, highly similar proteins can sometimes be observed with different annotated metal ions because of biological variation, metal substitution, experimental conditions, or differences in site occupancy. In this study, each metal‐binding site was labeled according to the metal ion annotated in the deposited structure, and such cases were retained if they satisfied the dataset filtering criteria. Therefore, the dataset does not enforce a unique canonical metal identity for each protein family; instead, it reflects experimentally observed metal‐binding annotations. To quantify the extent of such heterogeneity, we clustered the training proteins at high sequence similarity using MMseqs2 (95% sequence identity, 90% coverage) and found that 473 of 4,166 clusters contained differing metal compositions among highly similar members, whereas the rest 3,693 clusters showed a single metal composition. These heterogeneous clusters may represent true metal substitution at similar sites, distinct sites in homologous proteins, or annotation differences across deposited structures.

#### 4.1.4 Train/Validation/Test Split

Metal‐binding proteins were collected from BioLiP as of February 19, 2025. Entries available only in CIF format (with no corresponding PDB) and those determined by NMR spectroscopy were excluded. To reduce sequence redundancy, sequences were clustered at 30% identity using MMseqs2 [45, 46]:

mmseqs2 easy‐cluster <fasta> <Result> tmp –min‐seq‐id 0.3 –cov‐mode 0 –c 0.8 –s 8 –cluster‐mode 1

To prevent temporal and sequence redundancy biases, we first split chronologically (training/validation cutoff: 2019‐01‐01; validation/test cutoff: 2021‐03‐31) and then removed from validation/test any PDB that clustered with a training entry (Table S4). This ensured strict separation between training and evaluation data. Total counts of metals in our dataset in the training/validation/test set are reported in Table S5.

#### 4.1.5 Experimental Benchmark Sets

The held‐out test split described above was used as the main general PDB benchmark for evaluating metal‐site localization and metal‐type prediction on experimentally determined structures. For robustness analyses, this benchmark was further partitioned into X‐ray crystallography and cryo‐EM subsets according to the experimental method recorded in the PDB metadata.

#### 4.1.6 Model Structure Test Set

To evaluate performance on predicted protein structures, we created a benchmark using AlphaFold3 (AF3). Protein chains (chain A only) were extracted that contain metal‐binding sites with direct protein coordination (not ligand‐mediated). The extracted chains were modeled with AF3, and the predicted structures were aligned to experimental PDBs. We retained entries with global backbone RMSD ≤ 5 Å and local RMSD ≤ 3 Å within 5 Å of the metal site. This dataset supports evaluation under realistic modeling scenarios.

### 4.2 Benchmarking AF3 With Metals

For benchmarking AlphaFold3 (AF3) on metal‐binding sites, we used the Boltz‐1 test dataset [27]. In the dataset, protein chains were clustered at 40% sequence identity to avoid redundancy between training, validation, and testing. Training structures were drawn from PDB entries released before 2021‐09‐30 for AF3 and up to 2021‐03‐31 for PrOMet, while test candidates were restricted to structures released after 2023‐01‐13. For the AF3 metal benchmark in current work, the test set was re‐annotated with metal ions. We chose this benchmark because it is temporally separated from the both AF3 and PrOMet training data and additionally satisfies a <40% sequence‐identity relationship, enabling a fair comparison between AF3 and PrOMet.

#### 4.2.1 Negative Set

For the non‐binding discrimination task, we selected 316 proteins with no annotated bound metal ions from the Boltz‐1 test set. AF3 was prompted with a single Zn ion for each target to assess whether it spuriously places a metal into proteins that are not known to bind metal.

#### 4.2.2 Positive Set

For the site‐and‐type prediction task, we selected proteins containing a single metal‐binding site from the Boltz‐1 test set. This updated set was not restricted to single‐chain proteins; proteins containing multiple protein chains, DNA, or RNA were included, provided that the target contained only one annotated metal‐binding site. To improve representation of underrepresented metal classes, we additionally included cases from homo‐oligomeric proteins in which equivalent chains contained the same monometal site; in such cases, one representative chain‐site instance was selected. The final positive benchmark contained 82 cases in total: Ca (19), Mg (21), Zn (22), Fe (6), K (4), Mn (7), and Cu (3).

For this positive benchmark, AF3 was evaluated under two prompting strategies. In the All‐metal setting, all seven ions (Cu, Fe, Mg, Zn, Ca, Mn, and K) were prompted simultaneously in a single run. In the Per‐metal setting, one ion was prompted at a time, and AF3 was run independently for each of the seven candidate metals.

### 4.3 Model Architecture

#### 4.3.1 Grid Representation

We discretized the 3D space around protein complexes into grid points sampled from coordination spheres of atoms, defined by their van der Waals radii with a small expansion to capture regions where interactions and coordination can occur. Sterically clashing or redundant points were removed to obtain a compact, non‐overlapping set of nodes. Sampled points were located on protein surface only and were separated from neighbors by at least 0.6 Å.

#### 4.3.2 Random Forest for the Pruning Stage

Before constructing the graph, a Random Forest (RF) classifier was applied to prune grid points with low metal‐binding potential. All structural descriptors were computed around protein surface grid points. Each grid point was embedded with features summarizing the local chemical environment, geometric properties, and second‐shell context of putative metal‐binding sites; the full feature list is provided in Table S6.

1. Feature type 1: Residue‐ and ligand‐level coordination descriptors
  To characterize the first‐shell chemical environment, cumulative counts of nearby atoms were computed at five distance thresholds (2.5, 2.8, 3.0, 3.2, and 3.5 Å) centered on each grid point. Specifically, numbers were counted for (i) protein coordinating atoms (N, O, S), (ii) coordinating atoms from core metal‐binding residues (His, Cys, Asp, Glu), (iii) backbone carbonyl oxygen atoms, and (iv) ligand‐ and cofactor‐derived N, O, S, and Se atoms, negatively charged ions, and other heteroatoms. These descriptors quantify the coordination potential contributed by both the protein and any bound ligands or cofactors.
2. Feature type 2: Geometric and accessibility descriptors
  To assess steric clashes and hindrance, we included the distance from each grid point to the nearest nonpolar carbon atom and to the nearest backbone atom. Solvent exposure and cavity openness were captured by the relative solvent‐accessible surface area (rSASA), computed by first obtaining absolute SASA using the Shrake–Rupley algorithm (probe radius 1.4 Å) [47] and then normalizing by the maximal SASA of the same residue type. In addition, backbone preorganization of nearby core residues (His, Cys, Asp, Glu) [16] was encoded, which measures how closely a residue's backbone geometry matches backbone patterns typically observed at metal‐binding sites.
3. Feature type 3: Second‐shell descriptors
  To capture stabilizing interactions beyond the immediate coordination sphere, second‐shell features were defined in the 3.2–5.0 Å range around each grid point. These include the number of residues whose atoms fall within this shell, the number of core residues (His, Cys, Asp, Glu), and the number of backbone oxygen atoms. These second‐shell descriptors reflect hydrogen bonding, electrostatic stabilization, and structural support that help organize the metal‐binding environment.

The RF model outputs a metal‐binding probability for each grid point, and only grid points exceeding a predefined probability threshold were retained for subsequent graph construction. This pruning step substantially reduces computational cost while preserving grid points that are chemically and geometrically consistent with plausible metal‐binding sites.

The RF model was trained on protein monomers from the training set (multimers were excluded due to memory limits). To address strong class imbalance between binding and non‐binding grid points, a balanced RF was used that equalizes classes during bootstrap sampling. This preserved sensitivity to true binding regions while avoiding bias toward the abundant non‐binding class. We used BalancedRandomForestClassifier (imblearn v0.12.3) [16, 48] with default settings, specifying only random_state = 42 and n_jobs = −1 (100 estimators, Gini impurity, unlimited depth, bootstrap sampling, and automatic undersampling of the majority class).

#### 4.3.3 EGNN for the Optimization Stage

In the second stage, an Equivariant Graph Neural Network (EGNN) [26] was used to refine predictions within the candidate regions identified by the pruning step. Protein atoms and spatial grid points were jointly represented as nodes in a single graph, and each node was connected to its k nearest neighbors in Euclidean space (k = 16), forming a local interaction graph around putative metal‐binding sites (see Table S7).

Node features included the i) 3D Cartesian coordinates of each node, one‐hot encodings of ii) amino‐acid identity, iii) atom type, iv) secondary‐structure state [49], and v) chemically defined atom categories, as well as vi) solvent‐accessible surface area (SASA) [49, 50] and vii) partial atomic charge [51]. Grid nodes followed the same feature schema, with atom‐ or residue‐specific quantities set to neutral or default values when not applicable.

Edge features included i) one‐hot encoded inter‐node distance to count for geometric and chemical relations, ii) a categorical edge type (grid–grid, grid–protein, protein–grid, or protein–protein), and iii) a binary indicator of covalent bonding for protein–protein pairs. In addition, we introduced a iv) polarity‐based directional feature: for every edge, the angle θ was computed between the polarity vectors associated with the incident nodes (or, for atom–grid pairs, between the atomic polarity vector and the atom–grid displacement vector) and represented it as (sin θ, cos θ) (Figure S23). This polarity‐angle feature improved the model's ability to capture directional chemical interactions and consistently reduced validation loss compared to EGNN baselines without polarity information.

The model architecture consisted of three components (Figure S1): (1) an embedding layer, (2) an encoder, and (3) a decoder. The embedding layer was implemented as a single MLP that projects the input node features into a latent representation, and the encoder was composed of five stacked EGNN layers that integrate graph structure and 3D coordinate information. During EGNN message passing, only node features are iteratively updated, whereas Cartesian coordinates and edge features are provided as fixed inputs and are not updated. The decoder comprised two heads, each implemented as a five‐layer linear network: one head predicts the metal‐binding probability, and the other predicts the metal type. All layers used layer normalization, GELU activation, dropout (with a rate of 0.1), and residual connections. The model was trained with the Adam optimizer with a learning rate of 0.0001. Hyperparameters such as number of layers and the learning rate are determined by validation set performance.

#### 4.3.4 Loss Function

The network was trained with three loss terms: an occupation probability loss, a type loss, and an auxiliary type loss. The overall training objective is:

$$L=L_{occ}+L_{type}+L_{aux}$$

(1) Occupancy probability loss (L_occ_)

For each grid point i, a probability is predicted that any metal ion is present at that location. The target label is defined by a soft labeling scheme based on the distance d_i_ to the nearest metal ion:

$$y_{i}=f\;\left(d_{i}\right)=\exp\left(-\frac{d_{i}^{2}}{a}\right)$$

The scaling parameter *a* is chosen so that a point separated by 2 Å has a probability of 0.5:

$$f\;\left(2Å\right)=\exp\;\left(-\frac{2^{2}}{a}\right)=0.5\;\Rightarrow a=\frac{4}{\ln2}\;\approx 5.77\;\;{Å}^{2}$$

Thus, points closer than 2 Å to a metal ion receive labels larger than 0.5, and the probability decays smoothly to zero as the distance increases. The occupation head is trained with a standard binary cross‐entropy loss:

$$L_{occ}=-\frac{1}{N}\sum_{i=1}^{N}\left[y_{i}log{\hat{y}}_{i}+\left(1-y_{i}\right)\;\log\;\left(1-{\hat{y}}_{i}\right)\right]$$

where N is the number of training points. ${\hat{y}}_{i}$ is the predicted probability for grid point i that any metal ion is present.

(2) Type loss (L_type_)

For each point we also predict its type, i.e. one of the metal species or a None type. Let c_i_ denote the ground‐truth class for point i and *y_i_* the predicted probability for that class. To compensate for the strong class imbalance between different metal types and the None class, we use a class‐balanced cross‐entropy loss,

$$L_{type}=-\frac{1}{N}\sum_{i=1}^{N}w_{c_{i}}\log p_{i,c_{i}}$$

with class weights,

$$w_{c}=\frac{1}{M_{c}}$$

where M_c_ is the number of training points belonging to class c. Here $y_{i}\in \left\{0,\;1\right\}$ is the binary target for the true class of point i.

(3) Auxiliary type loss (L_aux_)

Finally, we add an auxiliary supervision signal that focuses on points located in the immediate vicinity of a true metal ion. For points within 2 Å of any metal, the exact metal type is predicted applying the same class‐balanced cross‐entropy as above:

$$L_{aux}=-\frac{1}{\left|S\right|}\sum_{i\in S}w_{c_{i}}\log p_{i,c_{i}}^{\mathrm{aux}},\;S=\left\{i:\;d_{i}\leq 2\;Å\right\}$$

This term encourages the model to learn precise metal‐type discrimination around actual binding sites.

The EGNN was trained on a system with 24 physical CPU cores (48 logical CPUs) and 4 NVIDIA RTX A5000 GPUs. Total training time was 2 days, 21 min, and 40 s.

#### 4.3.5 Clustering and Site‐Level Annotation

Grid‐level EGNN predictions are aggregated into site‐level annotations using DBSCAN (eps = 2, min_samples = 2) [26, 52, 53]. Neighboring high‐probability grid points are grouped into clusters corresponding to predicted metal‐binding sites. For each cluster, an overall binding probability is computed from the aggregated grid‐level scores and the site position is defined as the probability‐weighted mean of the grid coordinates within the cluster. The metal ion type of each site is assigned as the top‐1 class of the grid point with the maximum binding probability in the cluster. This post‐processing converts grid predictions into biologically interpretable site‐level calls and supports downstream evaluation at the site level.

#### 4.3.6 Inference Time

PrOMet runs by three steps: 1) feature extraction, 2) RF pruning, and 3) EGNN ranking. Using 1 CPU cores and a RTXA5000 GPU, inferencing a medium‐sized protein with 774 residues takes 1.26 secs (model‐loading time not counted).

### 4.4 Evaluation Metric

We treat metal‐binding site prediction as a binary classification problem at the site level. For a given decision threshold on the predicted site‐level probability, each candidate site is classified as either positive (metal‐binding) or negative (non‐binding). Let TP, FP, and FN denote the numbers of true positives, false positives, and false negatives, respectively.

The precision and recall are defined as

$$\mathrm{Precision}\;=\frac{TP}{TP+FP}$$

$$\mathrm{Recall}\;=\frac{TP}{TP+FN}$$

Precision measures the proportion of predicted positive sites that are actually true metal‐binding sites. Recall measures the proportion of true metal‐binding sites that are successfully identified by the model, i.e., among all truly metal‐binding sites, how many are recovered by the model.

To summarize the trade‐off between precision and recall with a single scalar, we report the F1‐score, defined as the harmonic mean of precision and recall:

$$F1-\mathrm{score}\;=\frac{2\cdot \mathrm{Precision}\;\cdot \mathrm{Recall}}{\mathrm{Precision}+\mathrm{Recall}}$$

A high F1‐score indicates that the model achieves both high precision and high recall simultaneously, while a low score suggests that either many predicted sites are false positives or many true sites are missed.

Because metal‐binding sites are relatively rare compared to non‐binding sites, the underlying data are highly imbalanced. In this setting, evaluating performance at a single, fixed decision threshold can be misleading. Instead, we consider the full precision–recall (PR) curve, which characterizes how precision and recall change as the decision threshold on the predicted probabilities is varied from 0 to 1. The area under the precision–recall curve (PR‐AUC) is then used as a threshold‐independent summary of model performance. We primarily report PR‐AUC as our main evaluation metric, alongside precision, recall, and F1‐score at representative thresholds.

### 4.5 Benchmarking Other Architectures

#### 4.5.1 Model for Pruning Stage

For the pruning‐stage comparison, we evaluated two additional baselines: LightGBM and MLP. Dataset and input features were kept identical to the original model. The LightGBM model was trained using LGBMClassifier from lightgbm (v3.3.5) with a binary objective and balanced class weights to address class imbalance. The model used 500 estimators, a learning rate of 0.05, 63 leaves, row subsampling of 0.9, and column subsampling of 0.9. The MLP baseline was implemented using MLPClassifier from scikit‐learn (v1.4.2). Before training, all input features were standardized using StandardScaler. The MLP consisted of three hidden layers with 128, 64, and 32 dimensions, respectively, and used ReLU activations, the Adam optimizer, L2 regularization with α = 10^−4^ and an initial learning rate of 10^−3^. Early stopping was enabled, using 10% of the training data as a validation split.

#### 4.5.2 Model for Optimization Stage

For the optimization‐stage comparison, we implemented two non‐equivariant graph baselines, GAT and GCN, by replacing the EGNN encoder while keeping the rest of the model unchanged. In both cases, node features were first projected from the 230‐dimensional input space to a 32‐dimensional latent representation using the same initial embedding module as in PrOMet. The GAT encoder consisted of five stacked single‐head graph attention layers, each followed by layer normalization, GELU activation, dropout, and residual connections, while maintaining a hidden dimension of 32 throughout. The GCN encoder likewise consisted of five stacked graph convolution layers with the same normalization, activation, dropout, and residual structure; to provide minimal geometric information, edges were weighted by a radial basis function (RBF) of the inter‐node distance when explicit edge weights were not available. In both baselines, the decoder architecture, training protocol, and evaluation setting were kept identical to those of the main PrOMet model, including Random Forest pruning at threshold 0.5, so that the comparison focused on the effect of encoder choice.

## Author Contributions

**Gangeun Park**: conceptualization, methodology, visualization, software, data curation, writing – review and editing, writing – original draft, formal analysis, validation. **Chaok Seok**: conceptualization, writing – review and editing, supervision, funding acquisition, investigation. **Hahnbeom Park**: supervision, methodology, writing – original draft, writing – review and editing, funding acquisition, conceptualization, validation.

## Conflicts of Interest

The authors declare no conflicts of interest.

## Code Availability

The source code used in this study is available at https://github.com/qkrgangeun/LigMet.

## Supporting information




**Supporting File**: advs78003‐sup‐0001‐SuppMat.pdf.

## Data Availability

The data that support the findings of this study are available from the corresponding author upon reasonable request.
