# Supplementary material for: PrOMet: Accurate and Robust Metal‐Binding Site and Type Prediction
Source: Adv Sci (Weinh). 2026 Oct 11:e78003. Online ahead of print. doi: 10.1002/advs.78003 (PMC13654862; doi:10.1002/advs.78003)
Supplement: Supplementary file 1 [file ADVS-9999-e78003-s001.pdf]

Supplementary Information to

PrOMet: Accurate and Robust Metal-binding site and Type Prediction

Gangeun Park[1], Chaok Seok[1,2*], Hahnbeom Park[3*]

[1] Department of Chemistry, Seoul National University, Republic of Korea
[2] Galux Inc, Republic of Korea
[3] Biomedical research division, Korea institute of Science and Technology, Republic of Korea

Table of Contents

- **Figure S1.** Architecture of the EGNN-based optimization model in PrOMet.
- **Figure S2.** Distribution of resolutions for training structures
- **Figure S3.** Case studies on metals binding on other than protein-only interfaces
- **Figure S4.** Per-metal precision–recall curves for metal-binding site detection
- **Figure S5.** Precision–recall curves for Zn-binding site localization on the X-ray-only, non-training-cluster subset
- **Figure S6.** Resolution distribution of the general PDB test set, split by experimental technique
- **Figure S7.** Backbone and sidechain RMSD of metal binding site between Alphafold2 models and crystal structures
- **Figure S8.** Distribution of metal–donor distances in experimental PDB structures and AlphaFold models
- **Figure S9.** Confusion matrix of metal type prediction with Crystal structure and AF3 model structure
- **Figure S10.** Distribution of AF3 pLDDT scores for Zn atoms placed in the negative set of non-metal-binding proteins
- **Figure S11.** Distribution of PrOMet scores for Zn atoms placed on the negative set of non-metal-binding proteins
- **Figure S12.** All high-confidence false positive PrOMet predictions on the negative benchmark
- **Figure S13.** All high-confidence false positive AF3 predictions on the negative benchmark
- **Figure S14.** Example of a likely AlphaFold3 false-positive metal prediction
- **Figure S15.** Precision–recall comparison of PrOMet with and without the Random Forest pruning stage
- **Figure S16.** Precision–recall curves and summary metrics for ablation experiments evaluating polarity vector removal and non-biological metal augmentation
- **Figure S17.** Zn-only training ablation of PrOMet against Metal3D.
- **Figure S18.** Ablation analysis of PrOMet model architecture and polarity-related features across metal classes.
- **Figure S19.** Effect of pruning-stage decision threshold on final prediction performance.
- **Figure S20.** Comparison of optimization-stage graph neural network architectures.

- Figure S21. Per-metal comparison of joint binding-site and metal-type prediction performance between the default PrOMet model and the no-metal-weighted-loss variant.
- **Figure S22.** Rationale and validation of the ≥3 protein-coordination threshold for identifying biological metal-binding sites
- **Figure S23.** Illustration of the polarity-vector feature between protein and grid nodes.

- **Table S1.** Summary of datasets and benchmark sets used in this study
- **Table S2.** Coordinate accuracy of predicted metal-binding site coordinates at method-recommended probability thresholds
- **Table S3.** Comparison of pruning-stage models across decision thresholds
- **Table S4.** Overview of dataset composition after chronological splitting and cluster-based redundancy filtering.
- **Table S5.** Distribution of metal-binding sites across training, validation, and test sets
- **Table S6.** Features for RandomForest training
- **Table S7.** Node and edge features for the GNN

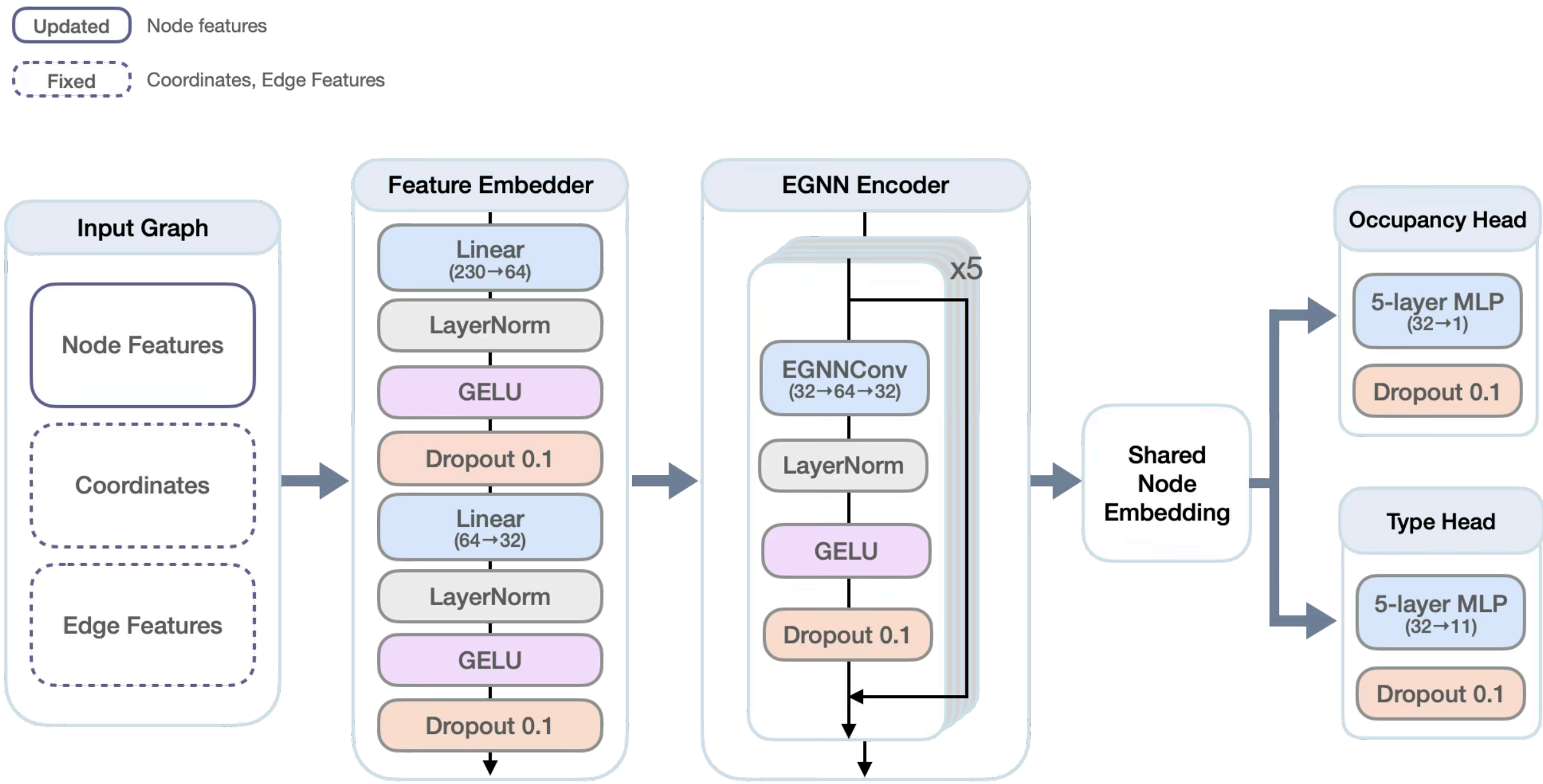

Figure S1. Architecture of the EGNN-based optimization model in PrOMet.
The model takes a protein–grid input graph comprising node features, Cartesian coordinates, and edge features. Raw node features are first projected by a two-layer feature embedder. The embedded node representations are then processed by five stacked EGNN encoder layers, each consisting of EGNN convolution followed by layer normalization, GELU, dropout, and a residual connection. During EGNN message passing, only node features are iteratively updated, whereas Cartesian coordinates and edge features are provided as fixed inputs and are not updated. The resulting shared node embedding is passed to two task-specific decoder heads: an occupancy head that predicts metal-binding probability and a type head that predicts metal types.

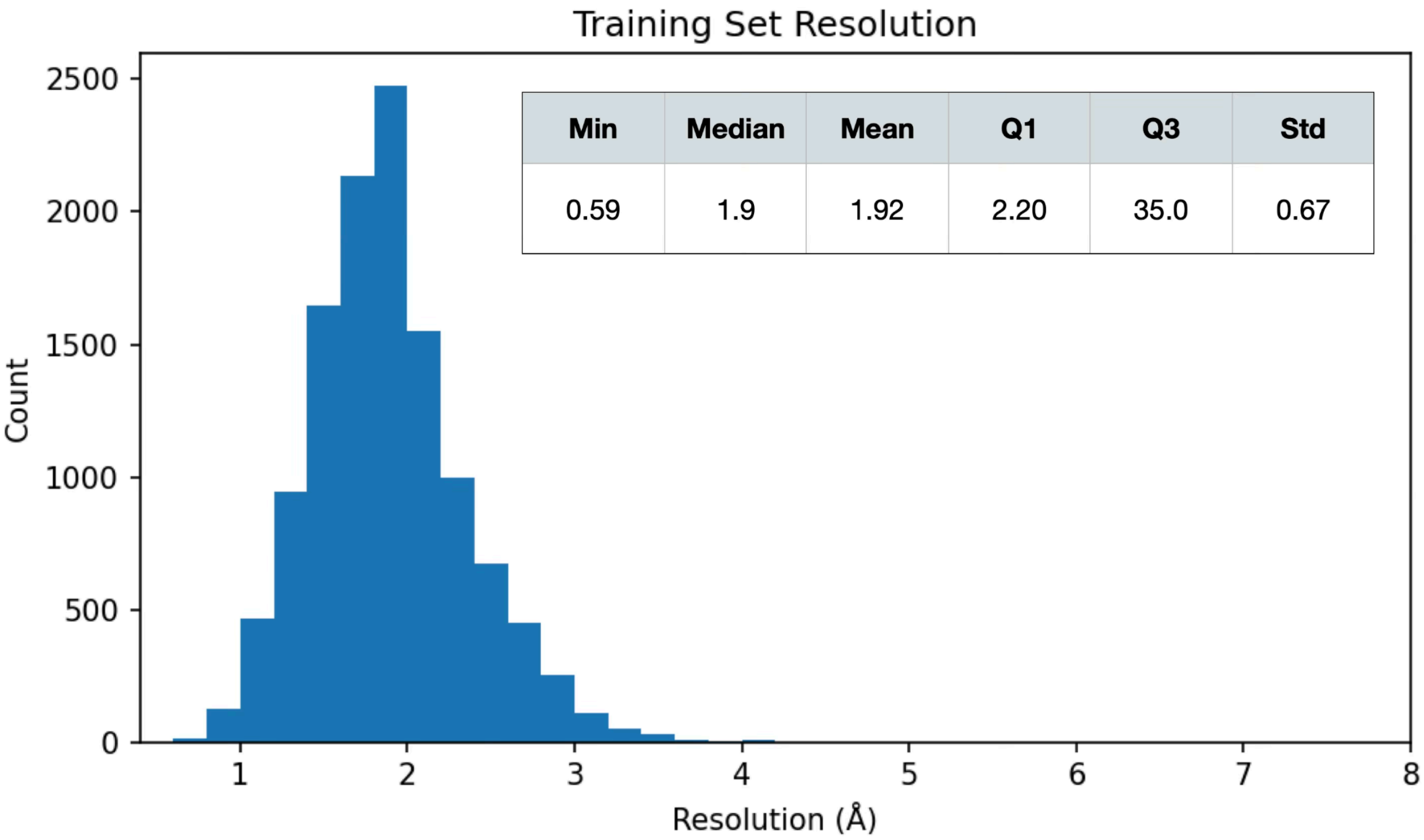

Figure S2. Distribution of resolutions for training structures. Histogram of the training set resolutions (Å); the inset table summarizes basic statistics (minimum, median, mean, first and third quartiles, and standard deviation).

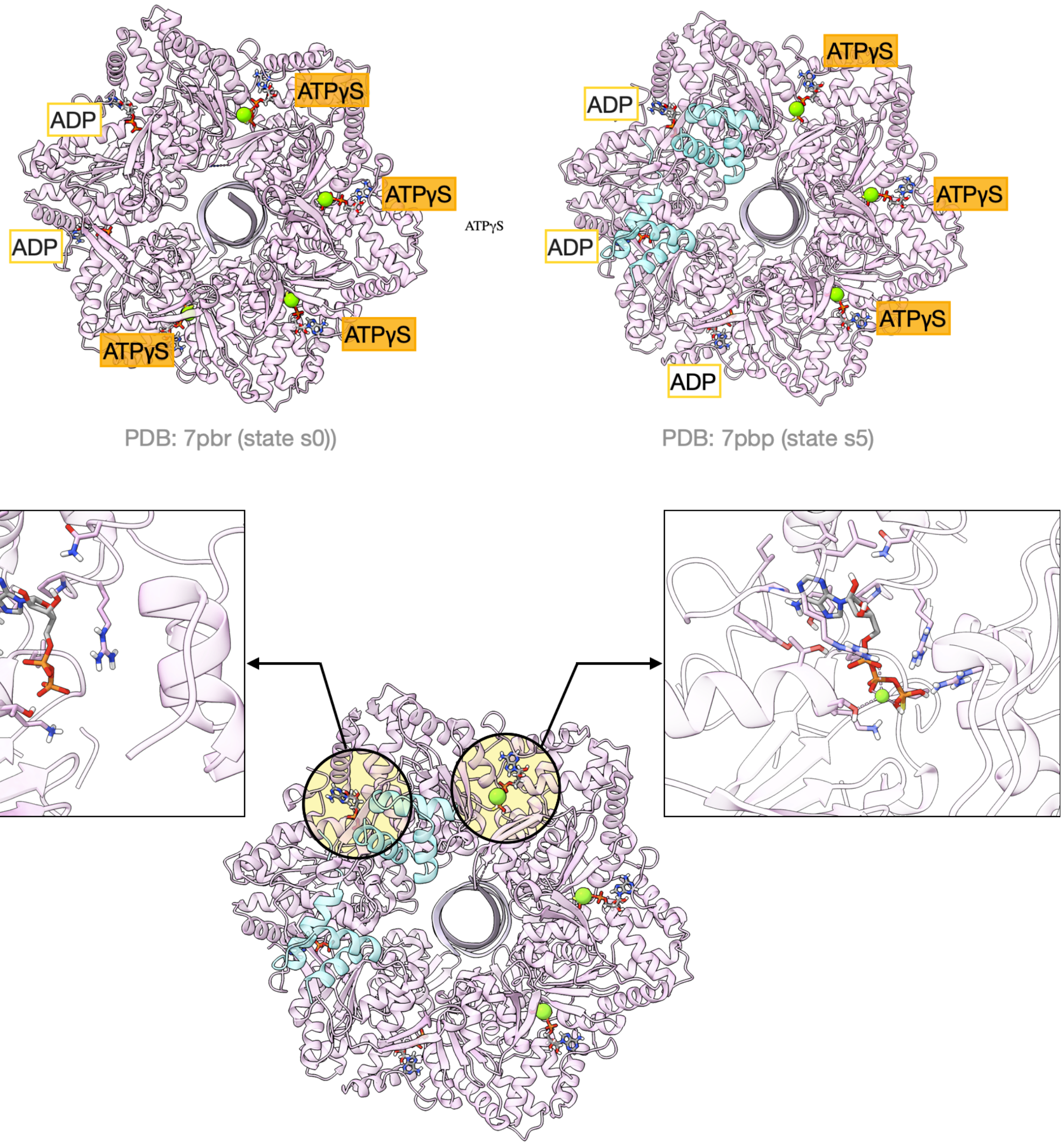

Figure S3. Case studies on metals binding on other than protein-only interfaces

In CASP15, several targets required identification of metal‑binding sites. As a representative example, we examined the RuvAB AAA$^+$ ATPase complexes (PDB 7PBR and 7PBP). These two states differ in their nucleotide composition around the RuvB hexamer—subunits bound to ATPγS tend to retain the catalytic Mg^{2+}, whereas ADP-bound subunits frequently lack Mg^{2+}. Our model recovered this ligand-dependent pattern in both structures: it localized Mg^{2+} sites to the nucleotide pockets of ATPγS-bound subunits and correctly predicted the absence of metal and its metal type at the corresponding ADP-bound pockets, matching the experimental occupancy pattern across the ring. Notably, performance was unchanged in 7PBP despite the presence of RuvA, indicating that the method is robust to assembly differences. Together, these results demonstrate that the model does not merely place metals at stereotyped motifs but conditions its predictions on the local ligand context, correctly capturing both position and presence/absence of Mg^{2+} at state-specific sites (evaluated at the site level using the ≤ 2.0 Å coordinate-tolerance criterion).

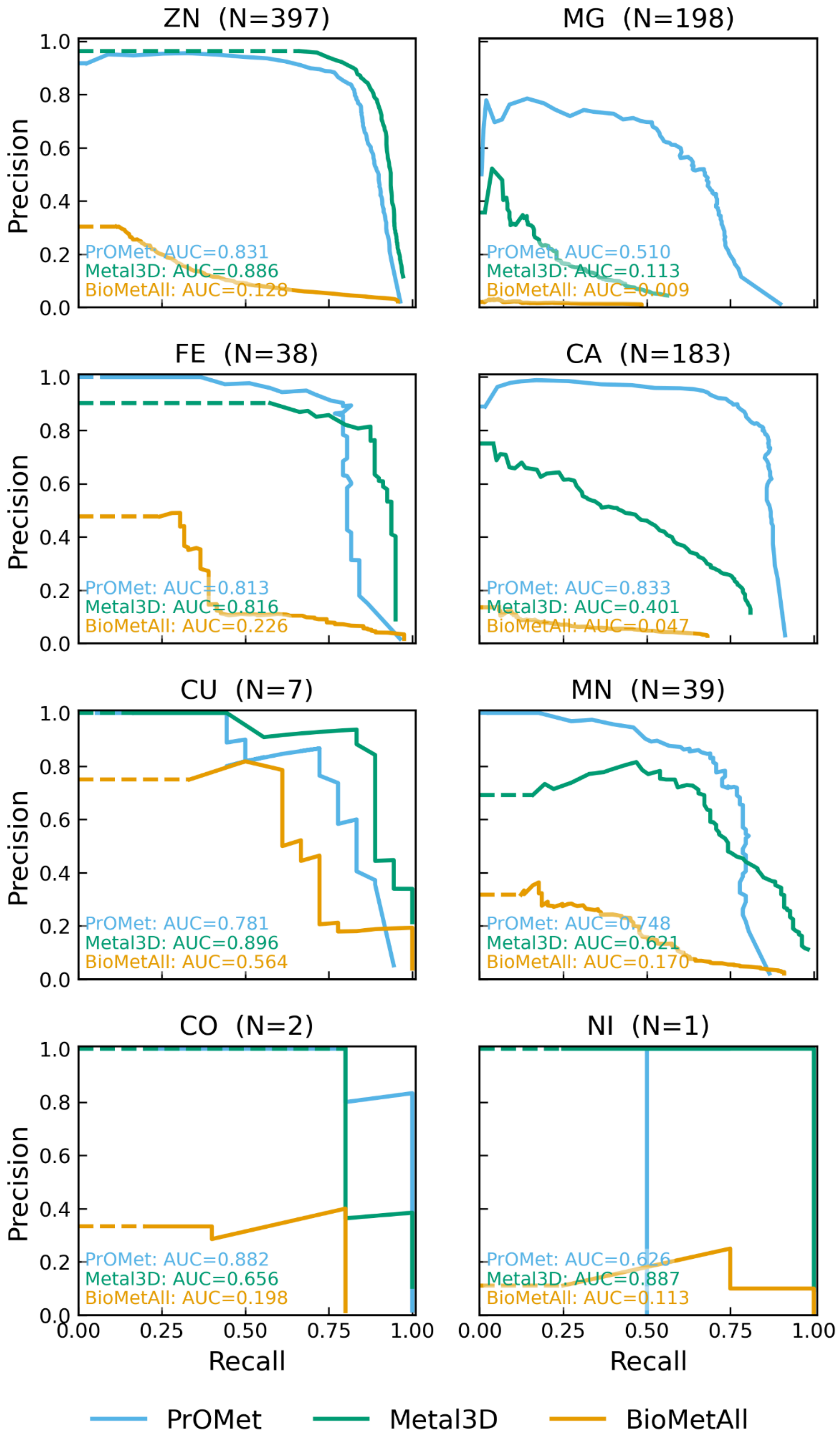

Figure S4. Per-metal precision–recall curves for metal-binding site detection. For each metal ion (Zn, Mg, Fe, Ca, Cu, Mn, Co, Ni), we constructed a dataset from our test set restricted to proteins containing only that metal species and evaluated whether the predicted binding sites were correctly localized to the true metal positions. Panels show precision–recall curves and PR-AUC values for PrOMet (blue), Metal3D (green), and

BioMetAll (orange); N denotes the number of protein structures in each metal-specific subset.

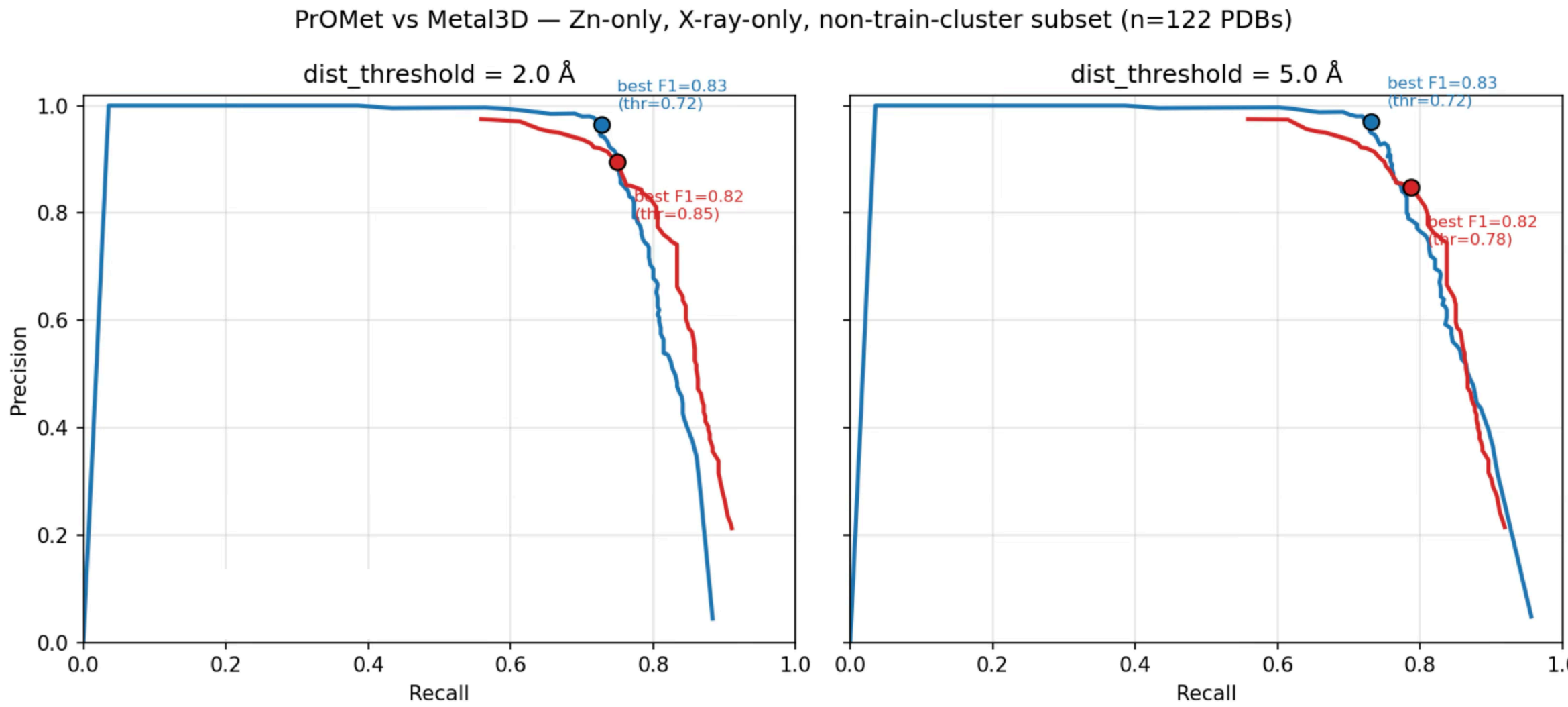

Figure S5. Precision–recall curves for Zn-binding site localization on the X-ray-only, non-training-cluster subset (n = 122 PDBs). PrOMet and Metal3D were evaluated using matching-distance cutoffs of 2.0 Å (left) and 5.0 Å (right). Circles indicate the operating points that maximize the F1 score for each method, with the corresponding best F1 scores and probability thresholds annotated. PrOMet achieved higher best F1 scores than Metal3D at both distance cutoffs while maintaining a favorable balance between precision and recall.

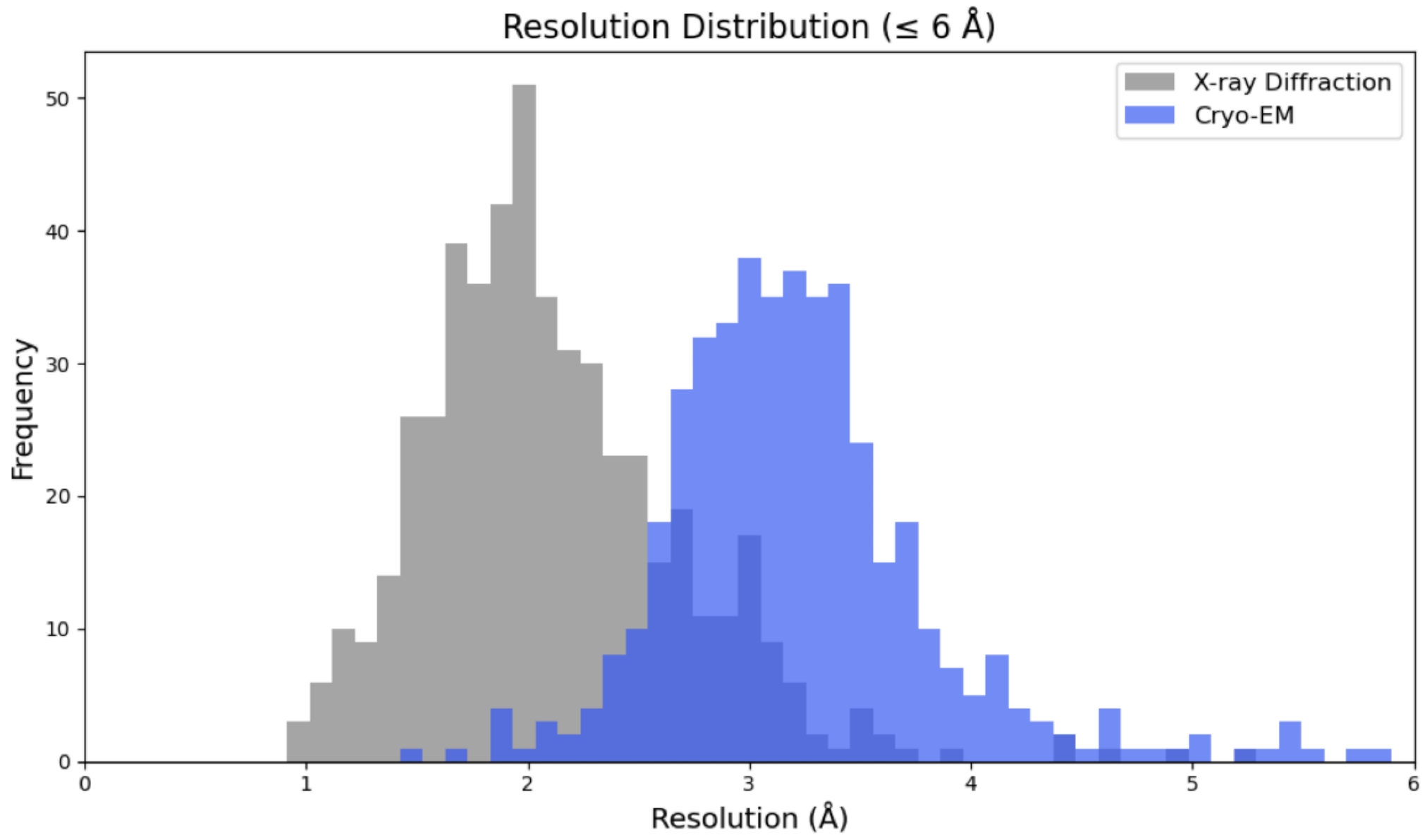

Figure S6. Resolution distribution of the general PDB test set, split by experimental technique. Histograms show the resolution (≤ 6 Å) of structures solved by X-ray diffraction (grey) and cryo-EM (blue), illustrating that cryo-EM entries in our dataset tend to have systematically lower (higher Å) resolution than X-ray structures.

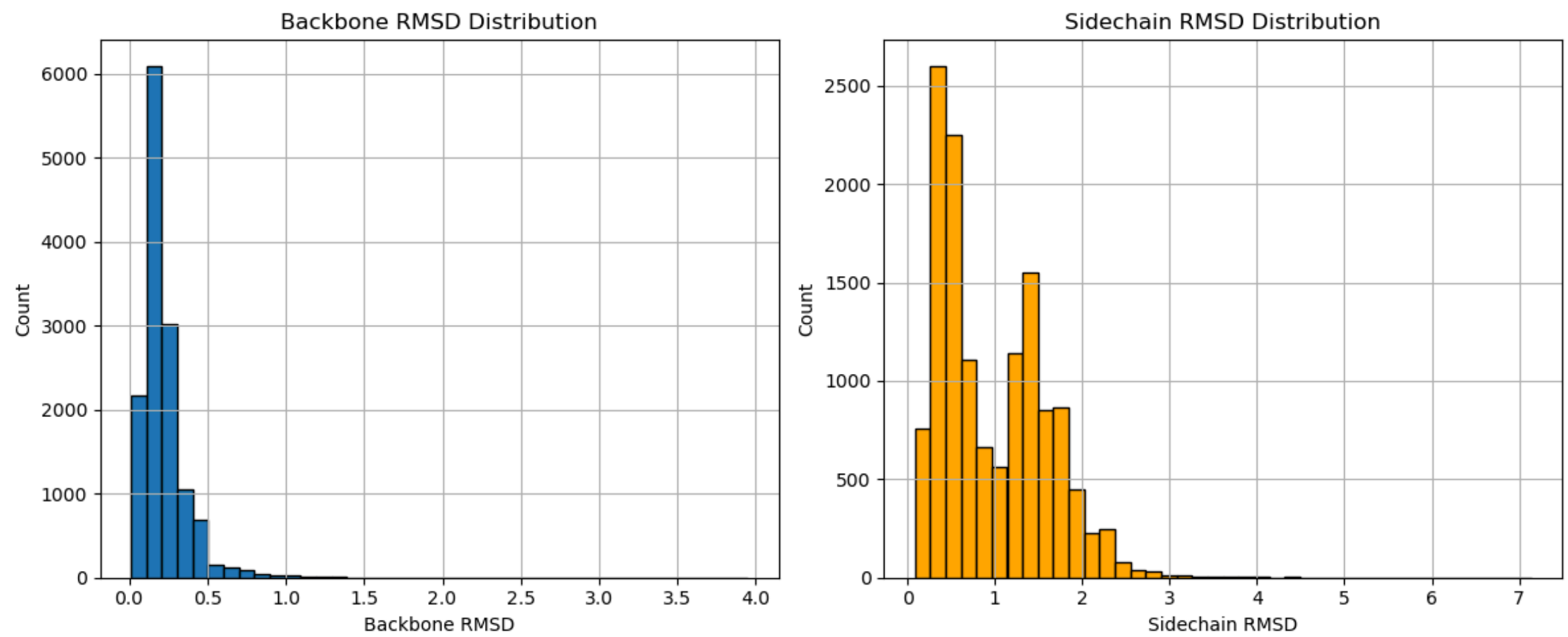

Figure S7. Backbone and sidechain RMSD of metal binding site between Alphafold2 models and crystal structures.

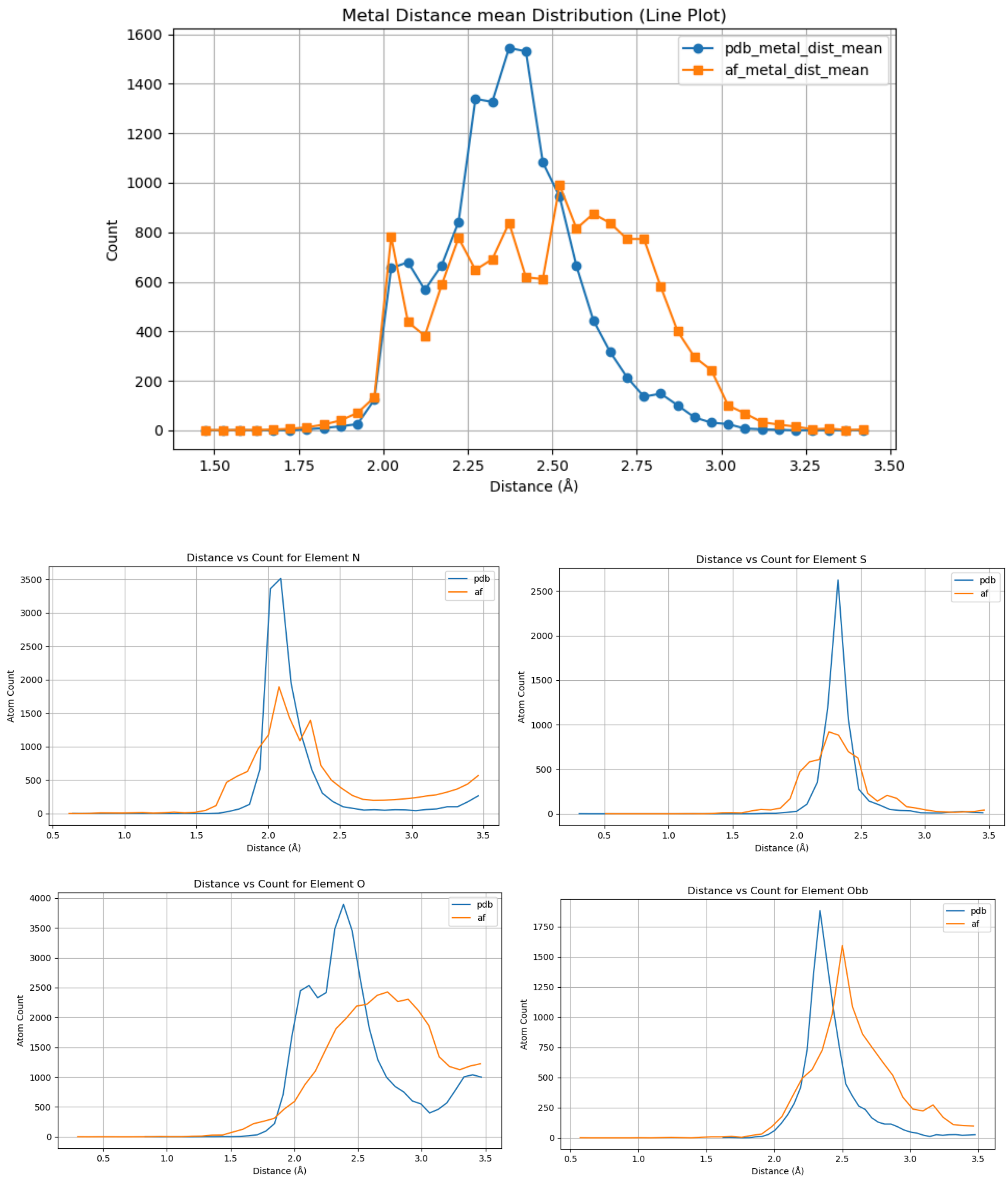

Figure S8. Distribution of metal–donor distances in experimental PDB structures (blue) and AlphaFold models (orange). While PDB structures show a sharp peak around 2.3–2.4 Å, AlphaFold models display broader and more variable donor–metal distances, reflecting larger deviations in modeled local geometries. This broadening is particularly driven by side-chain oxygen atoms, which frequently adopt less constrained conformations in the predicted models.

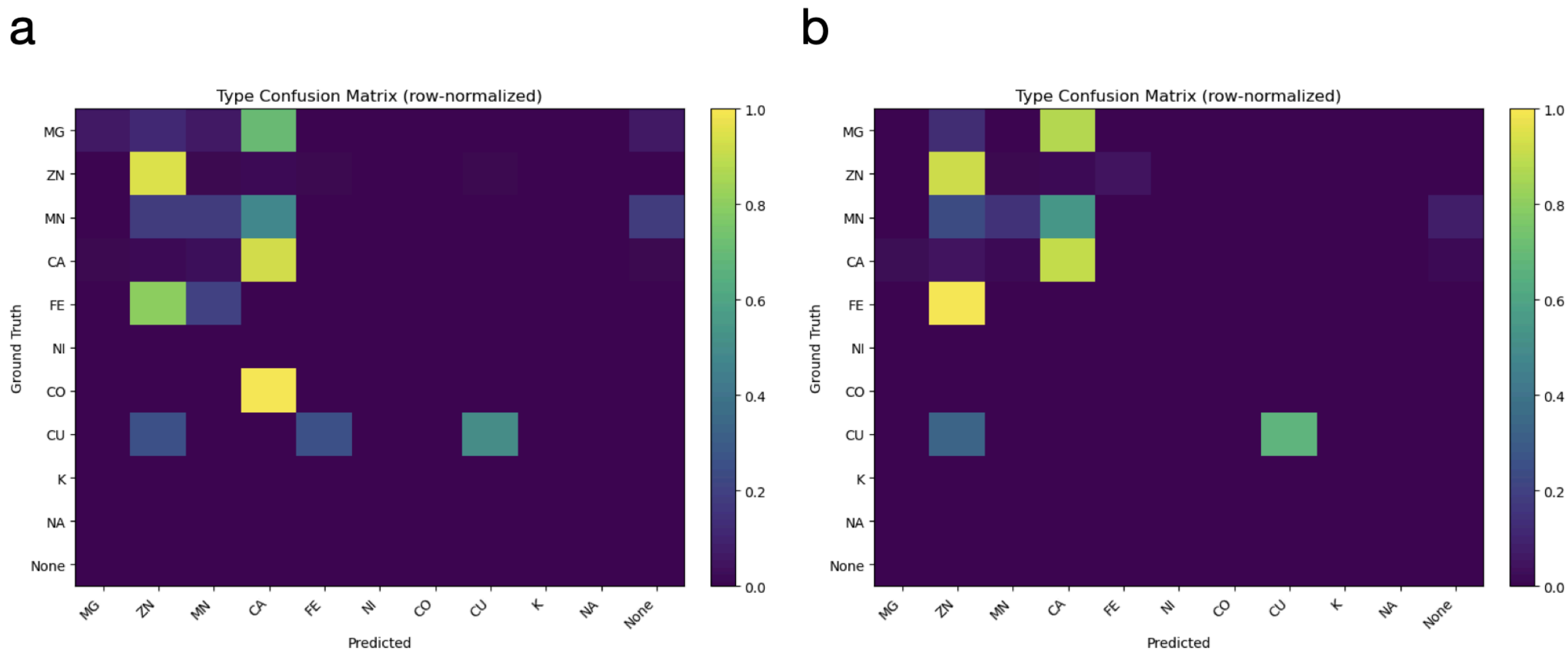

Figure S9. Confusion matrix of metal type prediction with Crystal structure (a) and AF3 model structure (b). They show similar patterns and no distinct difference between type prediction ordering.

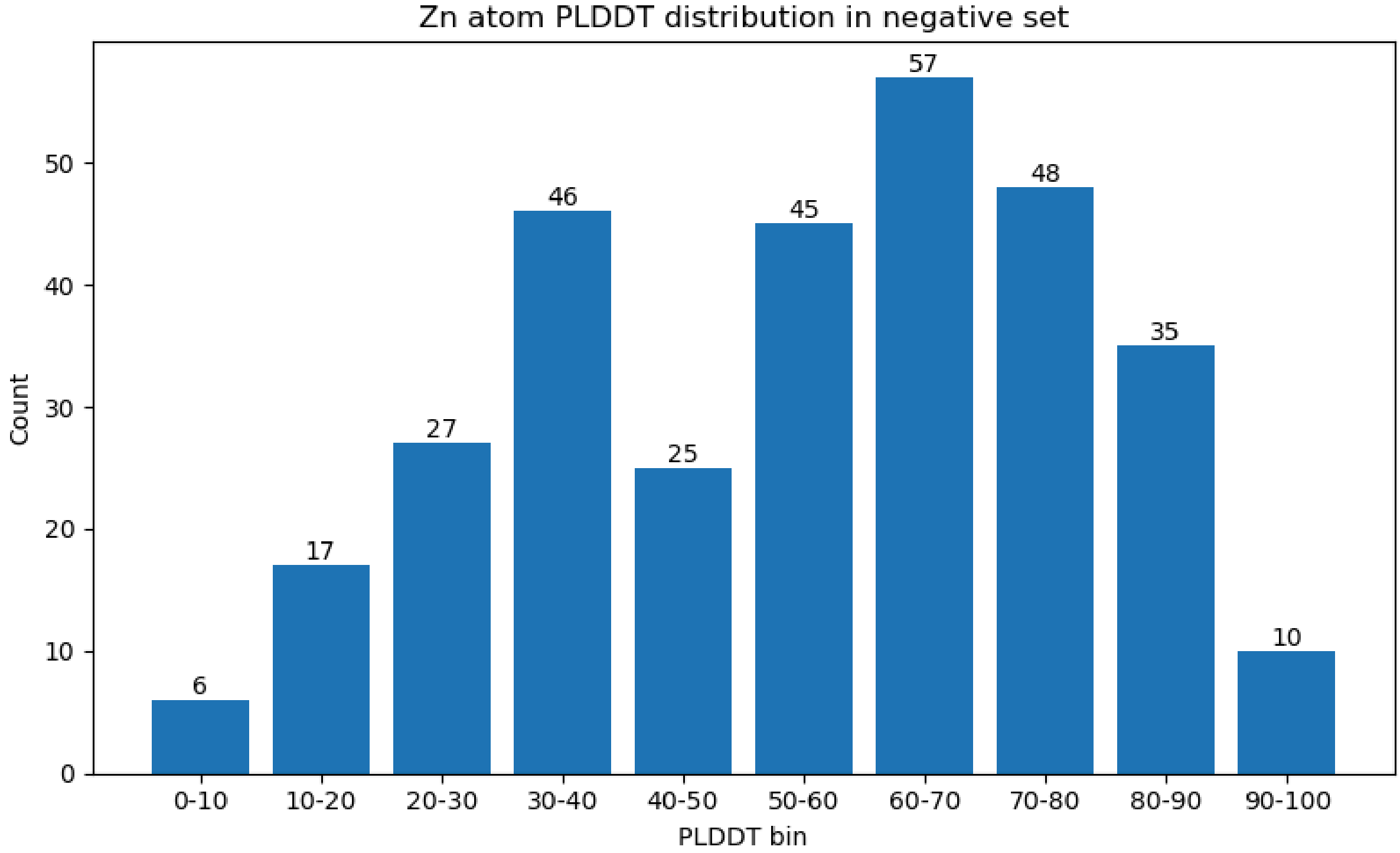

Figure S10. Distribution of AF3 pLDDT scores for Zn atoms placed in the negative set of non-metal-binding proteins. Even though these proteins have no annotated metal, AF3 frequently places Zn ions with moderate to high confidence (many with pLDDT > 60), indicating that the presence of a high-pLDDT metal atom alone is not sufficient to infer true metal binding.

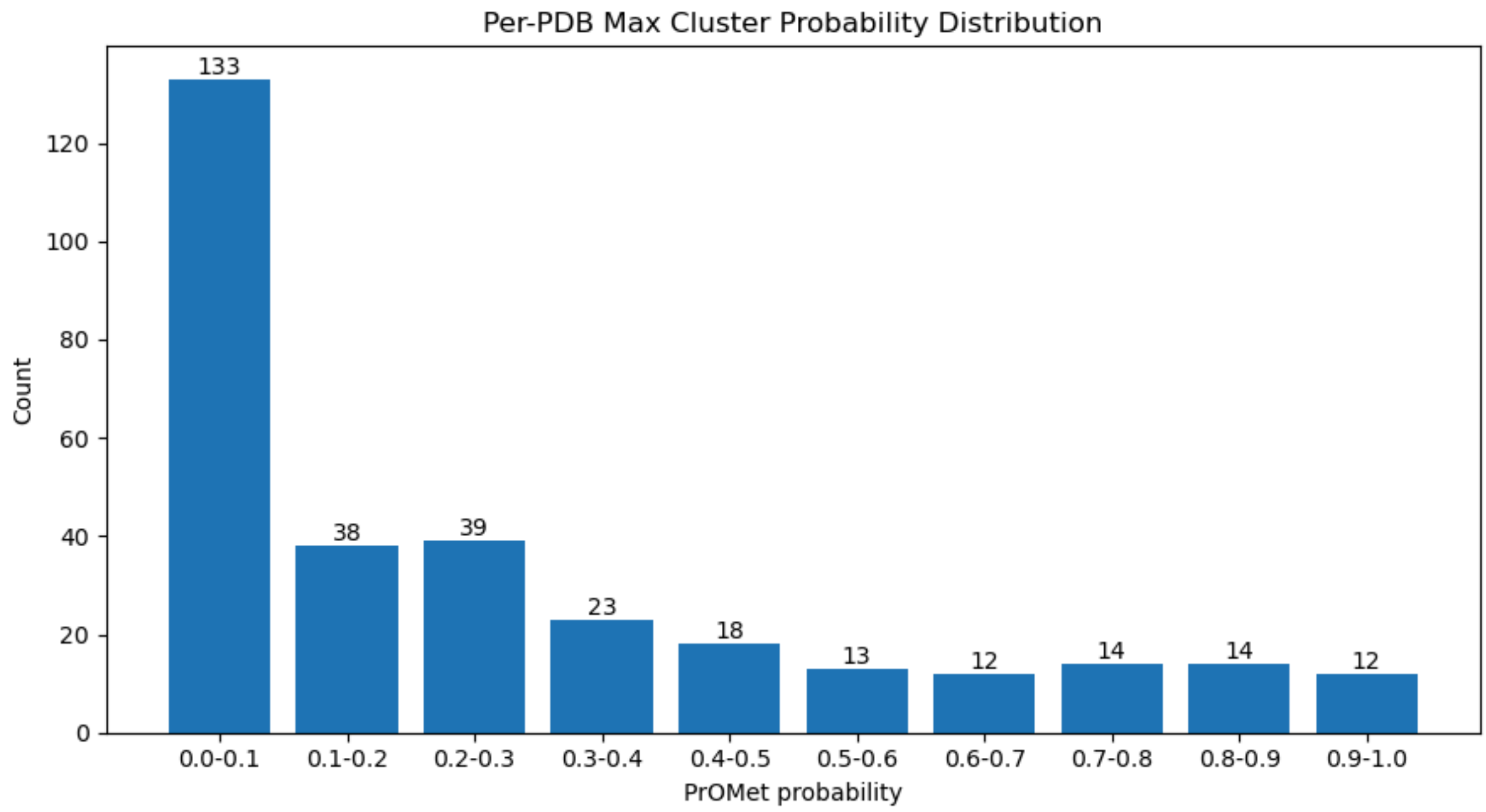

Figure S11. Distribution of PrOMet scores for Zn atoms placed on the negative set of non-metal-binding proteins. Distribution of the maximum predicted cluster probability per protein for 316 proteins with no annotated bound metal ions. Most proteins fall below the default prediction threshold of 0.5, indicating no above-threshold metal-binding prediction. The rate of proteins producing at least one predicted site varies on the probability threshold by 20.5%, 12.6%, 8.2%, and 3.8%, at cutoffs of 0.5, 0.7, 0.8, and 0.9, respectively.

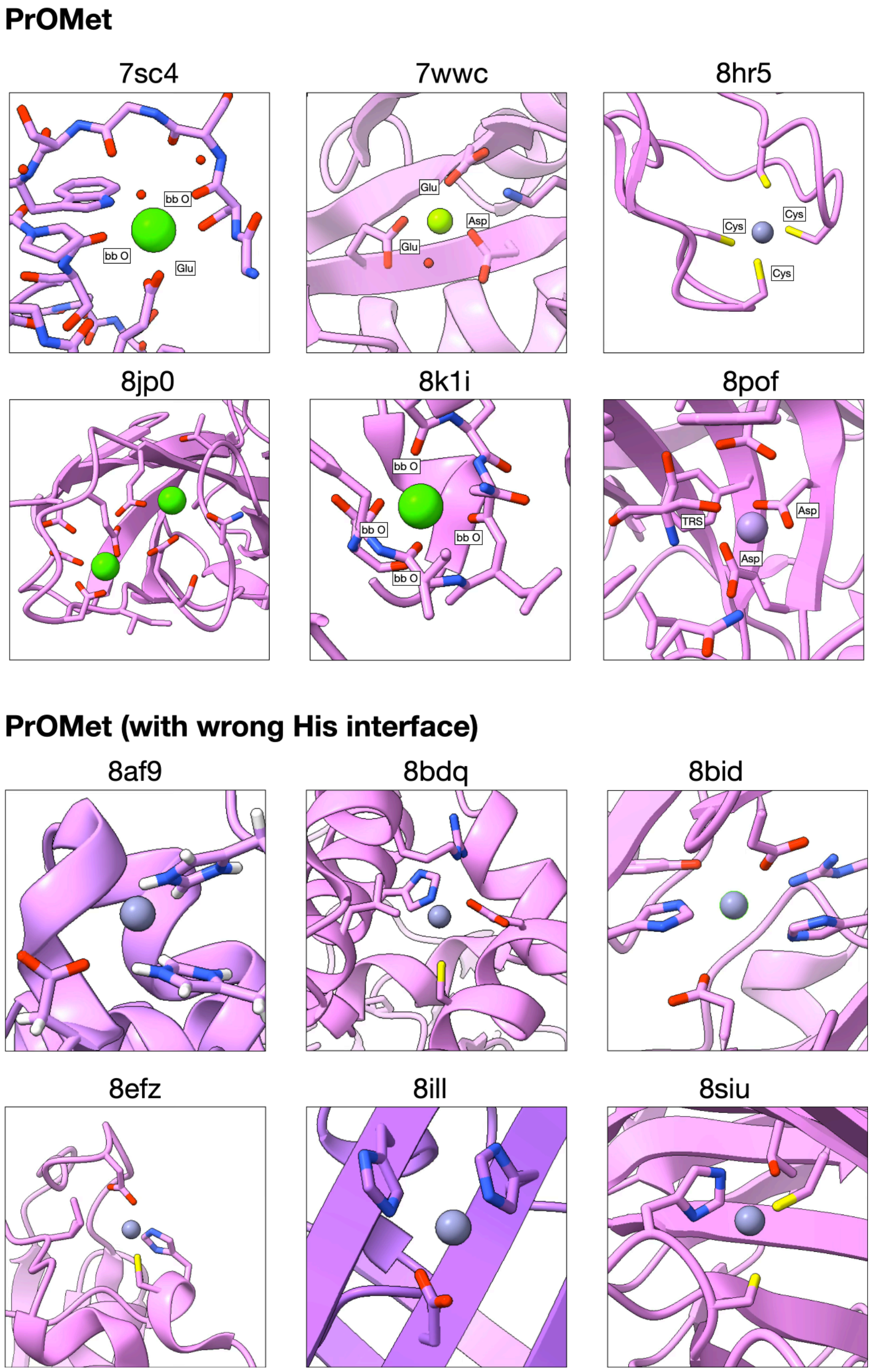

Figure S12. All high-confidence false positive PrOMet predictions on the negative benchmark. All 12 high confidence($p \geq 0.9$) false positive predictions with PrOMet are displayed. Top, many of these predicted sites still appear chemically plausible for metal binding, with local environments enriched in acidic residues, backbone carbonyl oxygens, or multiple cysteines/histidines. Bottom, examples of false positives potentially influenced by histidine tautomer ambiguity, where the chemically expected coordinating nitrogen would be NE2, but the local arrangement may instead favor or confuse ND1-related coordination geometry.

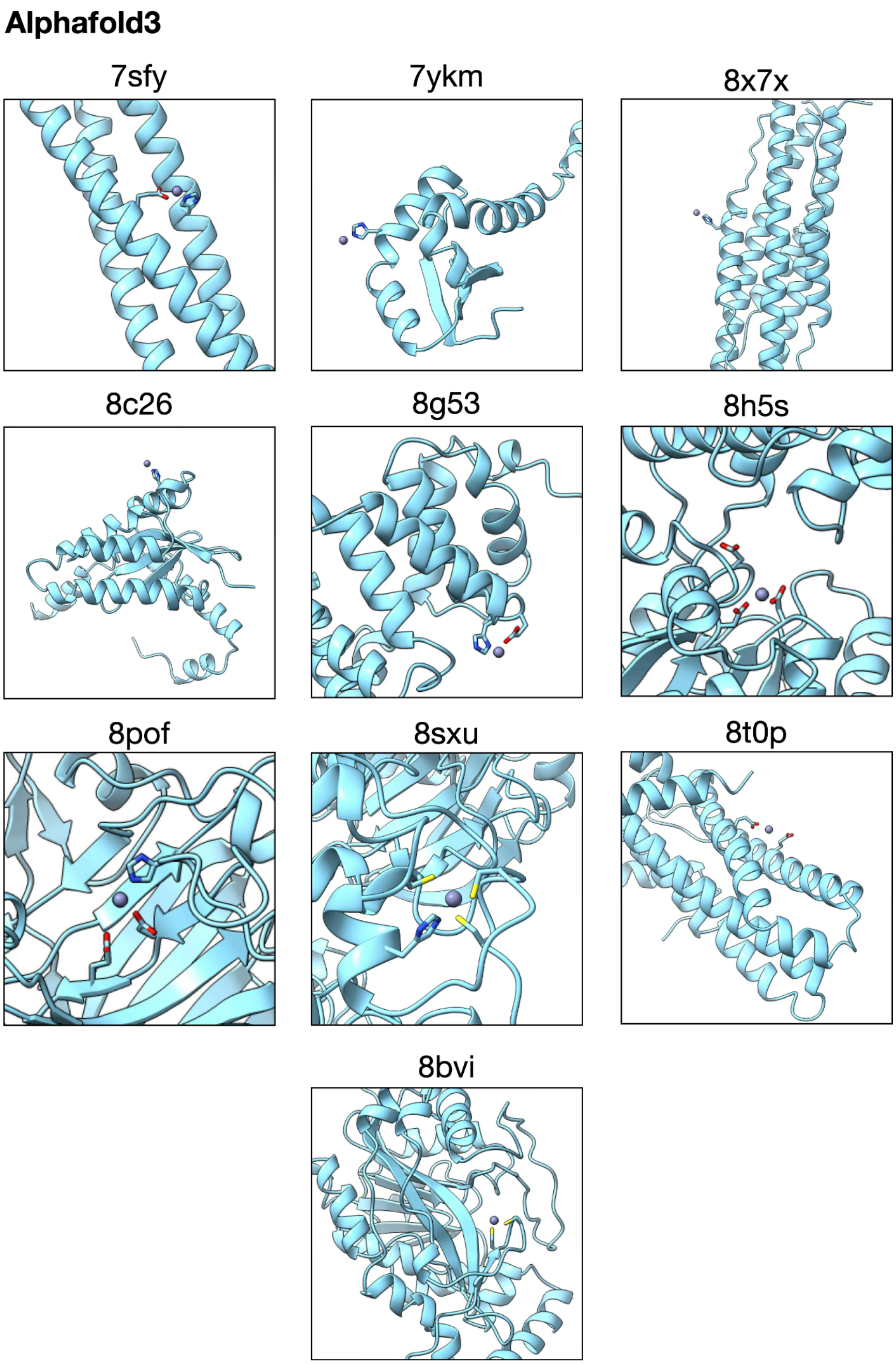

Figure S13. All high-confidence false positive AF3 predictions on the negative benchmark. All 10 high-confidence false-positive AF3 predictions, defined as cases with metal pLDDT $\geq$ 0.9. In several cases, the predicted metal placements appear to have limited chemical support for metal binding in the apo state, including insufficiently coordinated environments(only 1 or 2 coordinations) or local structural arrangements that do not strongly justify metal binding without ligand or additional stabilizing interactions.

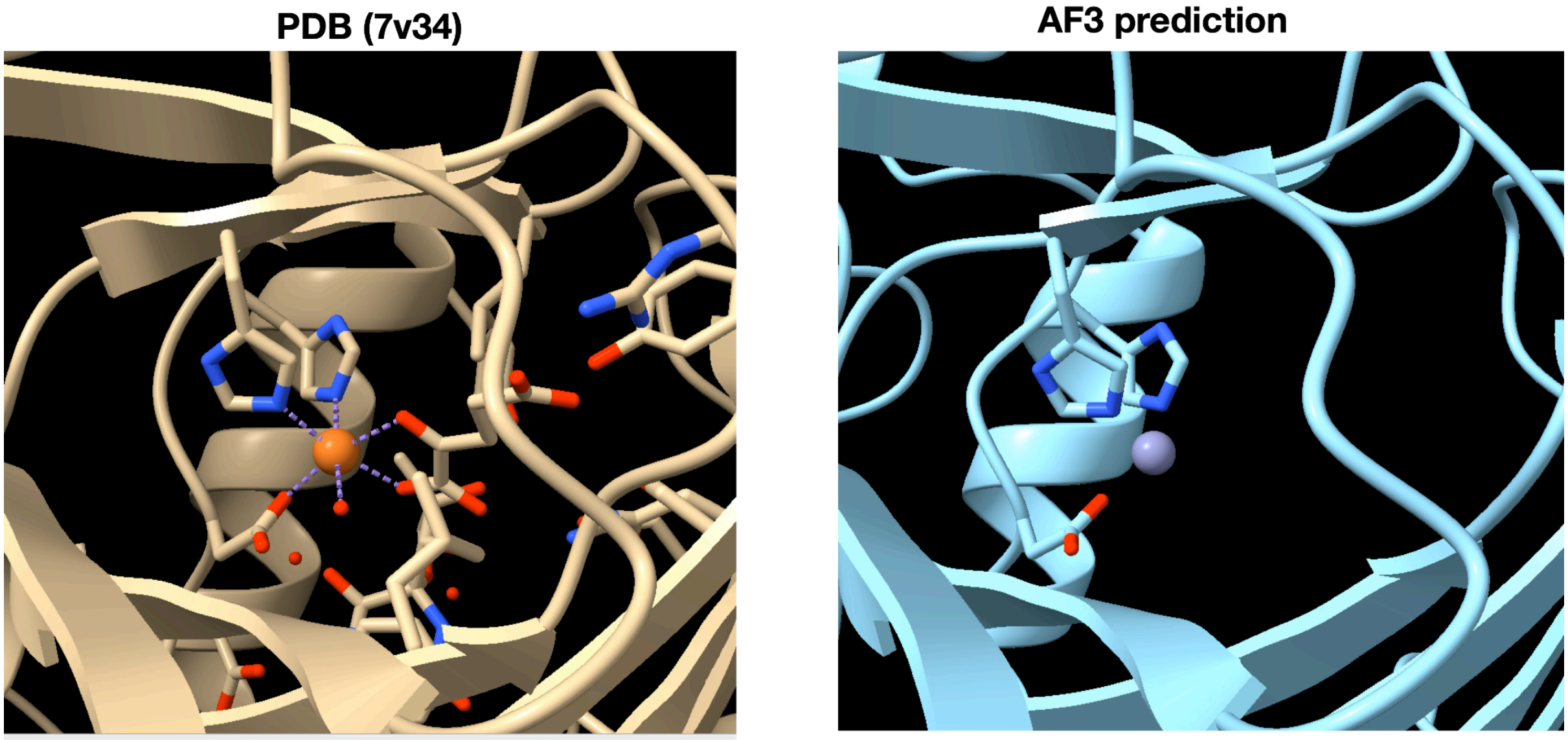

Figure S14. Example of a likely AlphaFold3 false-positive metal prediction. Left) deposited PDB structure in which metal binding is experimentally supported by a bound ligand, despite a histidine orientation that appears inconsistent with the chemically expected coordinating nitrogen (NE2). Right) AlphaFold3 prediction for the corresponding ligand-free structure. AlphaFold3 reproduced a similar histidine orientation and predicted metal placement at the same site with high confidence, even though the ligand-dependent coordination environment is absent. This example is consistent with the idea that some AlphaFold3 false positives may reflect reproduction of local patterns in deposited structures rather than chemically plausible de novo metal recognition.

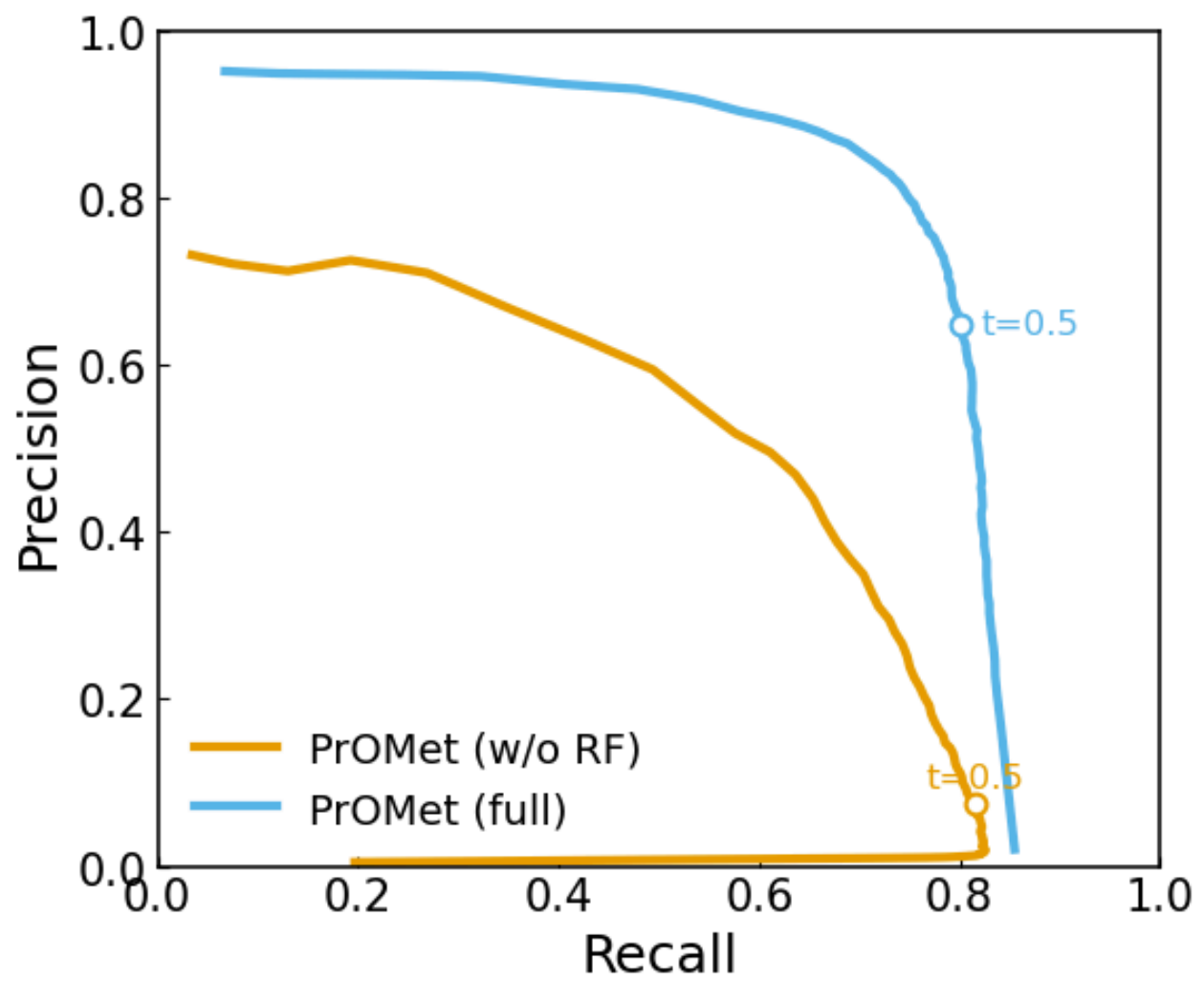

Figure S15. Precision–recall comparison of PrOMet with and without the Random Forest pruning stage. At threshold t = 0.5, the ablated model without Random Forest achieves low precision (7.75%) despite high recall (81.4%), whereas the full PrOMet maintains both high precision (65.6%) and recall (80.0%), highlighting the critical role of the pruning stage in improving prediction quality.

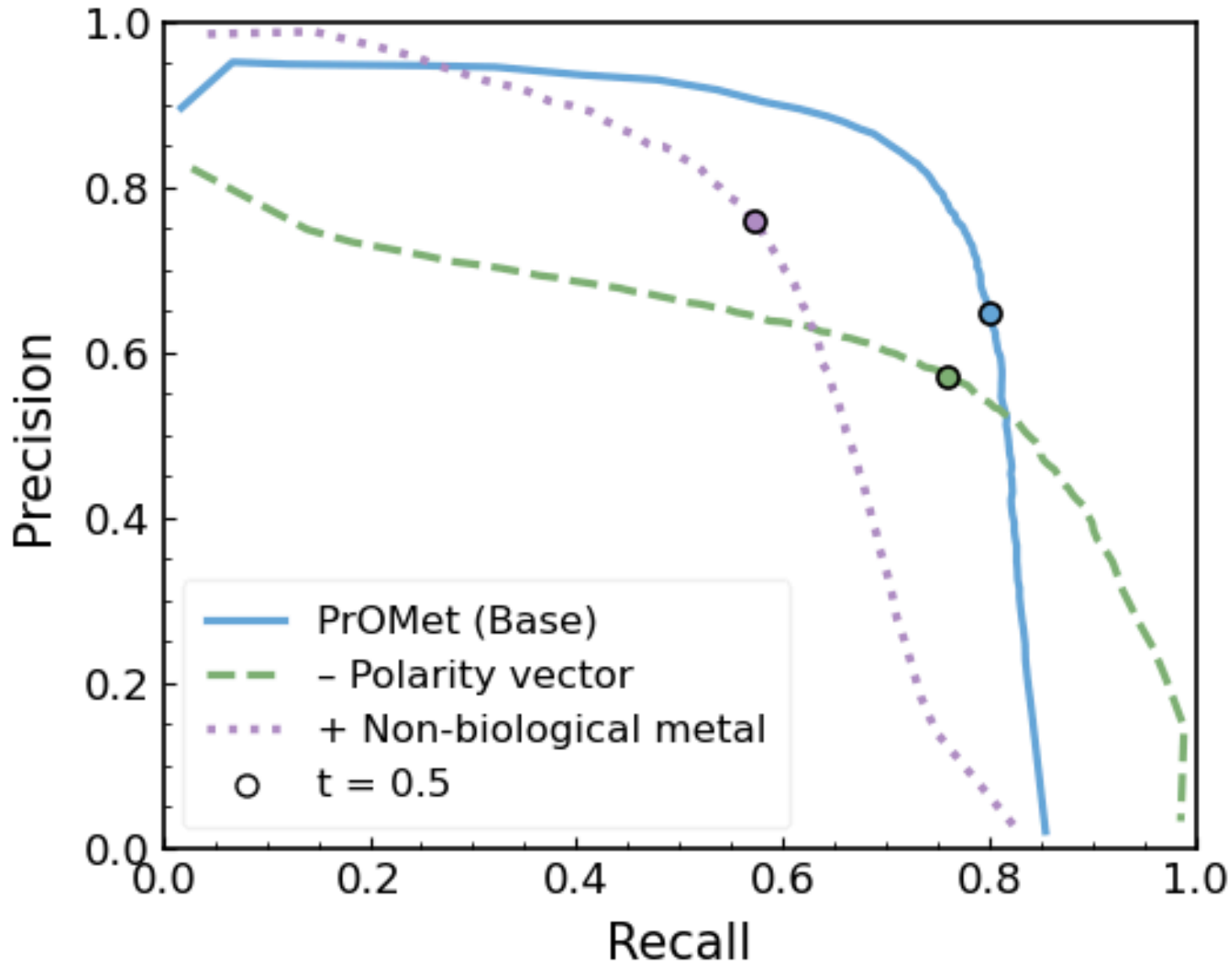

Model	Precision	Recall	F1 score	PR-AUC
Base	0.650	**0.800**	**0.717**	**0.739**
- Polarity vector	0.572	0.760	0.653	0.609
+ Nonbio Metal	**0.760**	0.572	0.653	0.605

Figure S16. Precision–recall curves and summary metrics for ablation experiments evaluating polarity vector removal and non-biological metal augmentation. The base model shows the highest overall PR performance, while removing the polarity vector substantially decreases precision across nearly all recall ranges. Including non-biological metals increases precision but at the cost of markedly reduced recall. Threshold-specific performance at $t = 0.5$ is highlighted with markers, and corresponding precision, recall, F1, and PR-AUC values are summarized in the table below.

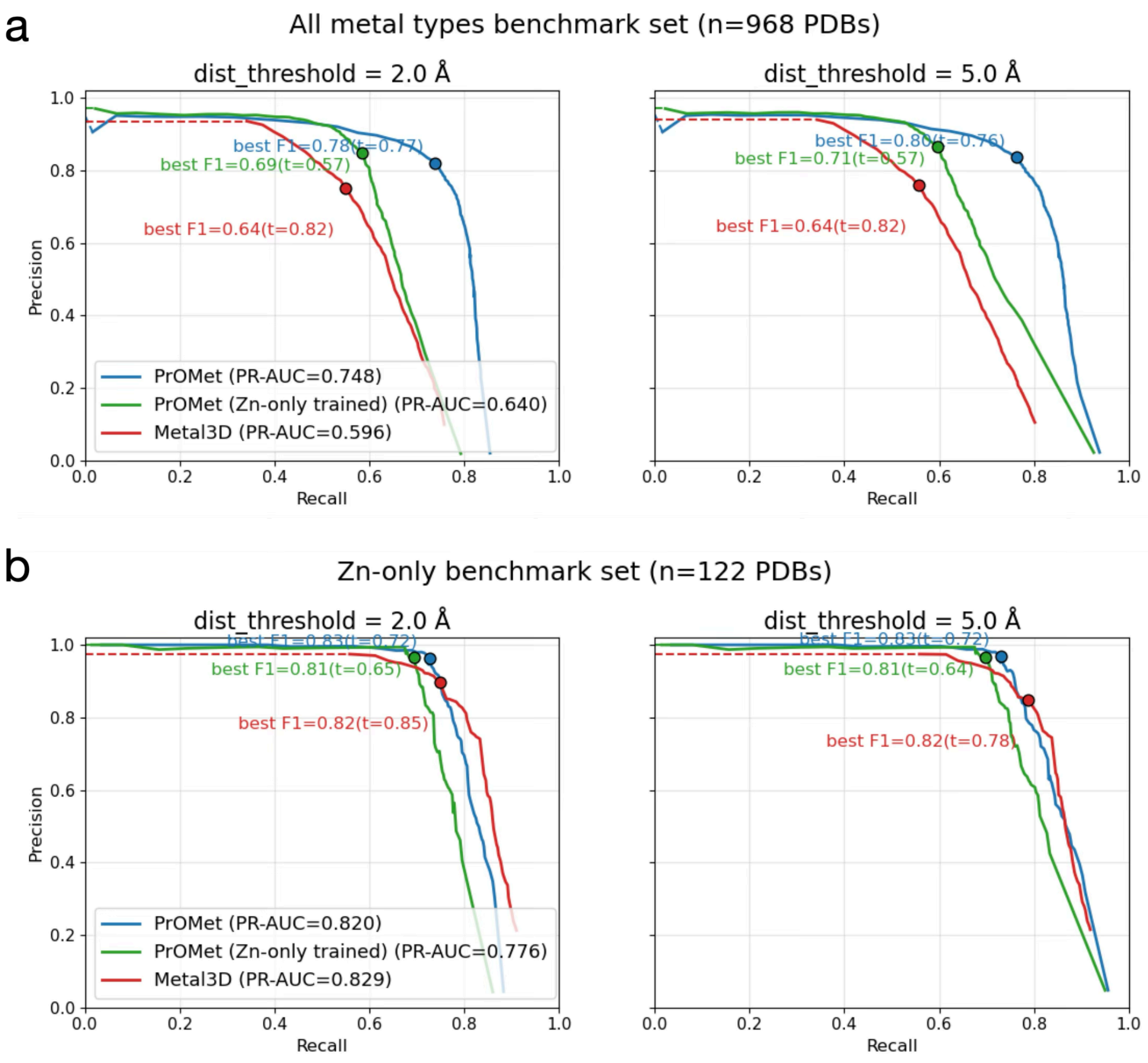

Figure S17. Zn-only training ablation of PrOMet against Metal3D. Precision–recall curves compare PrOMet (blue), PrOMet trained with Zn only, and Metal3D (red) using metal-site matching distance thresholds of 2.0 Å (left) and 5.0 Å (right). (A) Performance of the full PrOMet model PrOMet trained on Zn only and Metal3D on the complete multi-metal test set (*n* = 968 PDBs) (B) The full PrOMet model and Metal3D were evaluated on a scope-matched subset containing only Zn-bound X-ray structures that were not assigned to training-related sequence clusters (*n* = 122 PDBs).

a

b

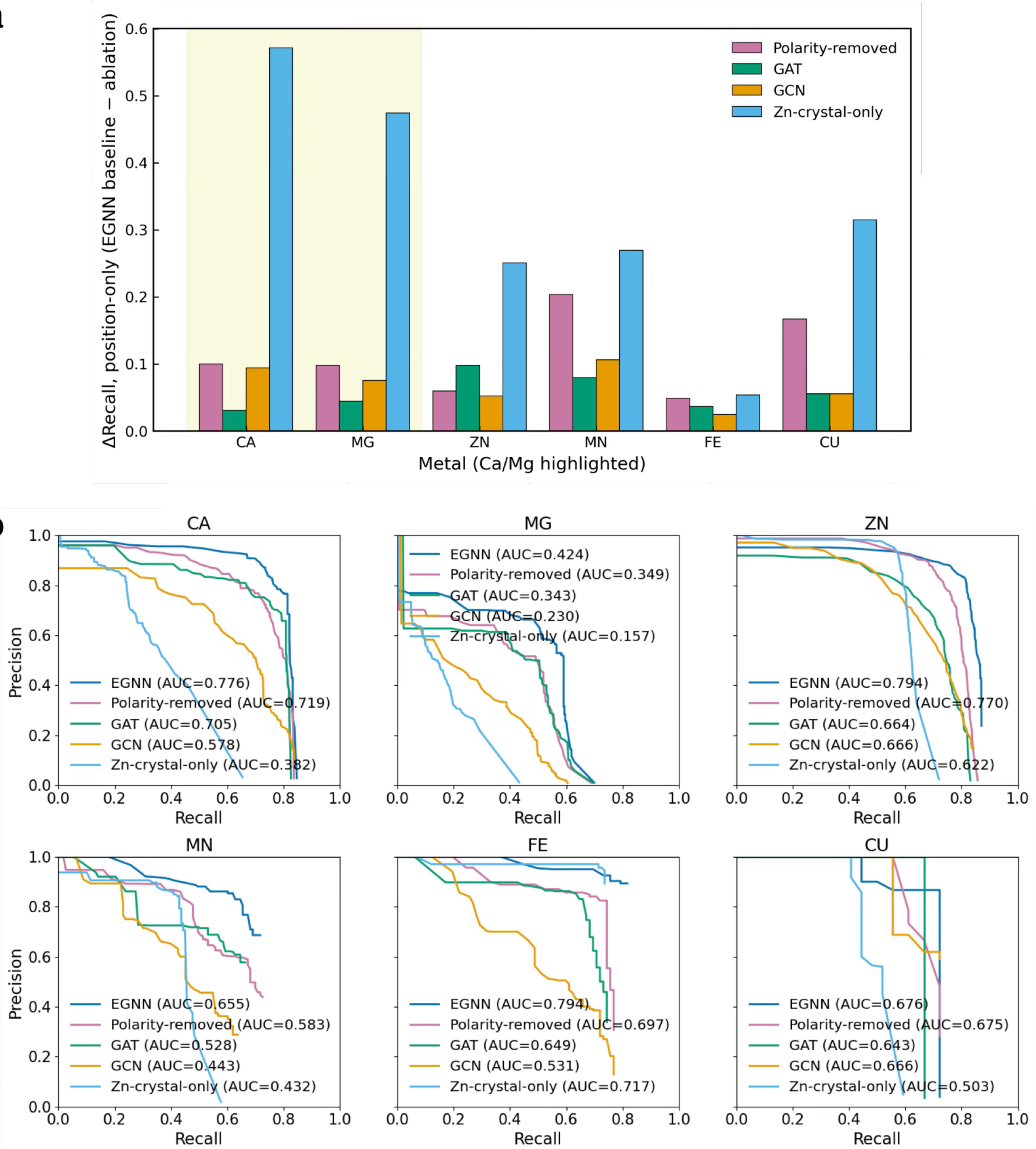

Figure S18. Ablation analysis of PrOMet model architecture and polarity-related features across metal classes. (a) Recall difference between PrOMet and ablation model. Higher values mean large decreases in recall upon ablation. (b) Precision–recall curves are shown separately for Ca, Mg, Zn, Mn, Fe, and Cu binding-site prediction.

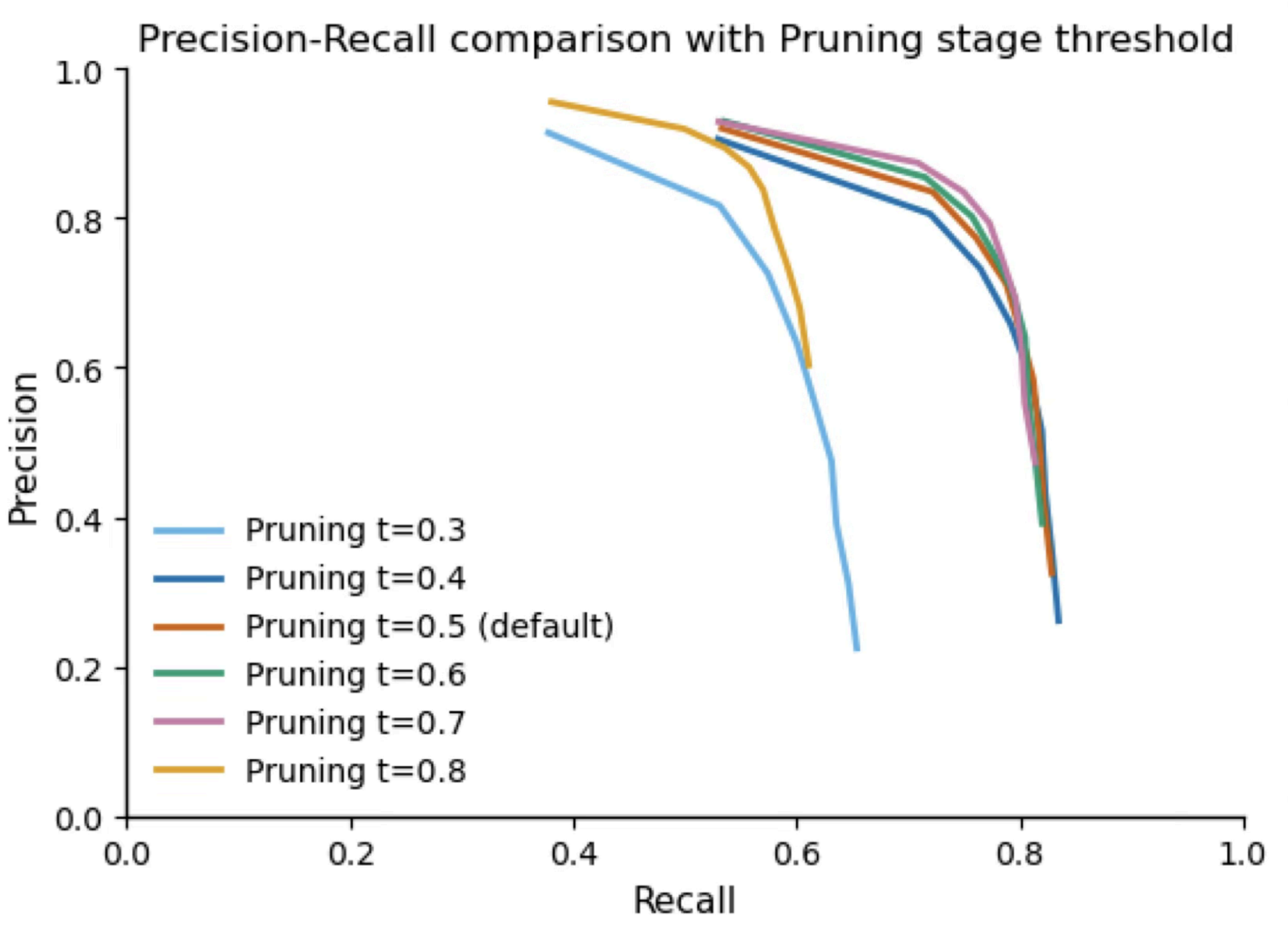

Pruning threshold (t)	Precision	Recall	F1
0.3	0.554	0.616	0.583
0.4	0.590	**0.808**	0.682
0.5	0.652	0.800	0.718
0.6	0.704	0.793	0.746
0.7	0.751	0.783	**0.767**
0.8	**0.838**	0.570	0.679

Figure S19. Effect of pruning-stage decision threshold on final prediction performance. Precision–recall (PR) curves for the full PrOMet pipeline obtained using optimization-stage thresholds t=0.3,0.4,0.5,0.6,0.7, and 0.8 while keeping the optimization-stage threshold fixed at 0.5. The accompanying table summarizes precision, recall, and F1-score at each threshold. Performance remained relatively stable across the range t=0.4–0.7, indicating that the pruning stage is not overly sensitive to the exact choice of threshold within this interval. Although the default threshold was set to 0.5, the highest F1-score was observed at t=0.7. These results suggest that thresholds in the range 0.4–0.7 can be selected depending on the desired precision–recall trade-off for a given application.

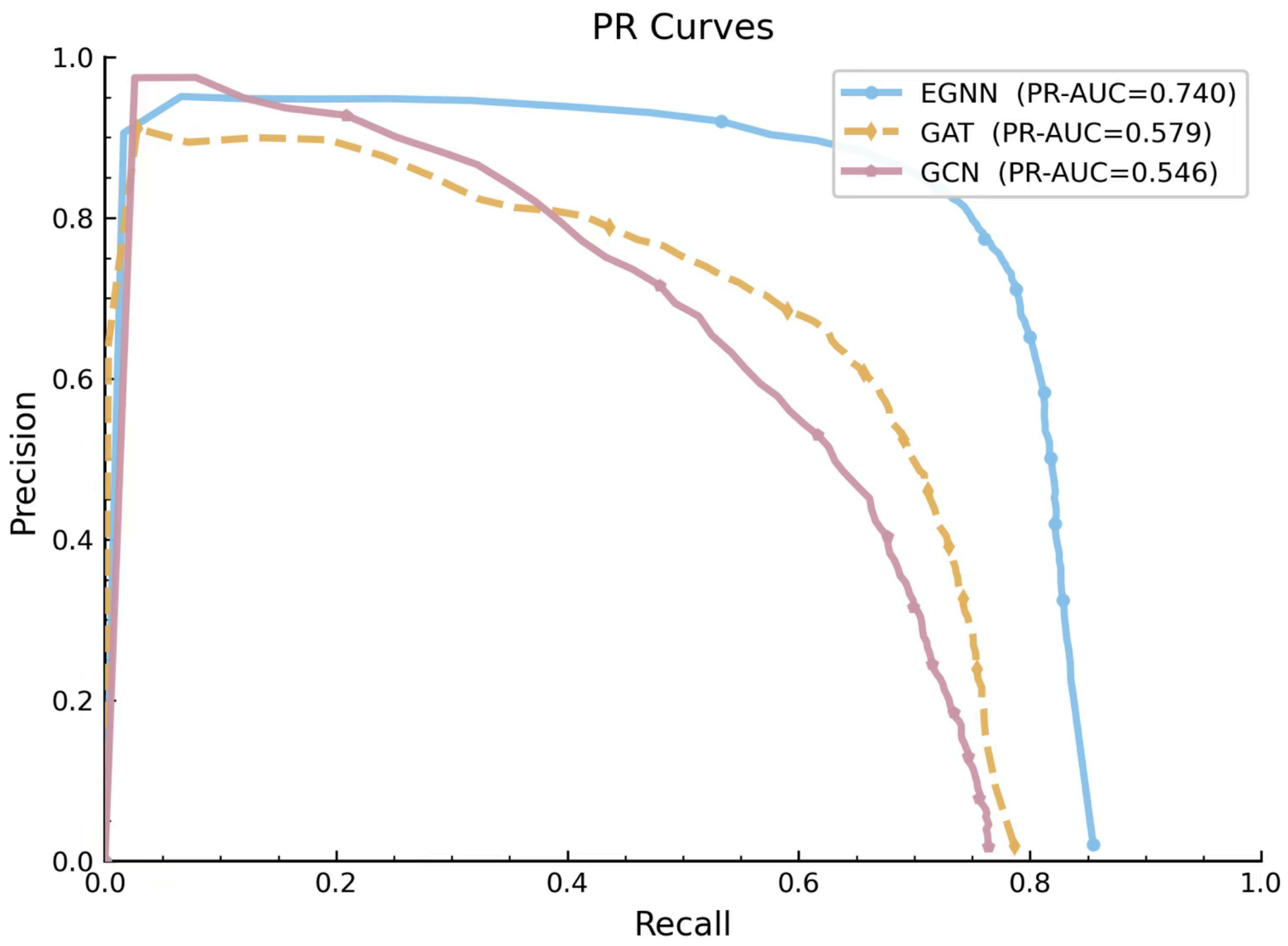

Model (t=0.5)	Precision	Recall	F1-score
EGNN	**0.650**	**0.796**	**0.716**
GAT	0.544	0.716	0.618
GCN	0.328	0.727	0.453

Figure S20. Comparison of optimization-stage graph neural network architectures. Precision–recall (PR) curves for EGNN, GAT, and GCN on the optimization-stage task. EGNN achieved the highest PR-AUC (0.740), followed by GAT (0.579) and GCN (0.546). The accompanying table reports the precision, recall, and F1-score of each model at a decision threshold of 0.5.

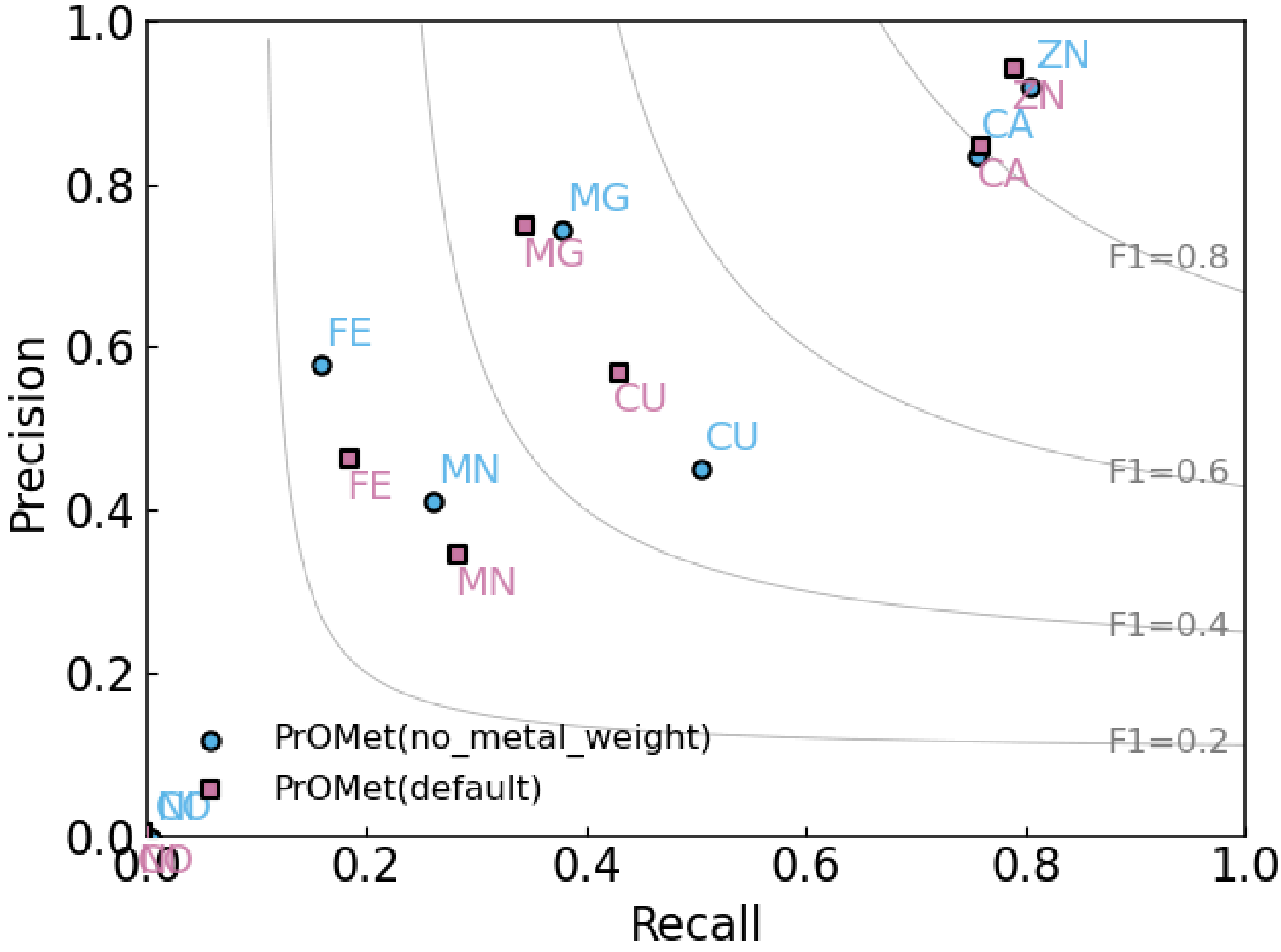

Figure S21. Per-metal comparison of joint binding-site and metal-type prediction performance between the default PrOMet model and the no-metal-weighted-loss variant. Per-metal precision, recall, and F1-score comparison on the test set for the default PrOMet model (squares) and PrOMet without metal-type loss weighting (circles). The overall trend remains similar, indicating that the weighting mainly affects the precision–recall balance rather than the qualitative performance pattern.

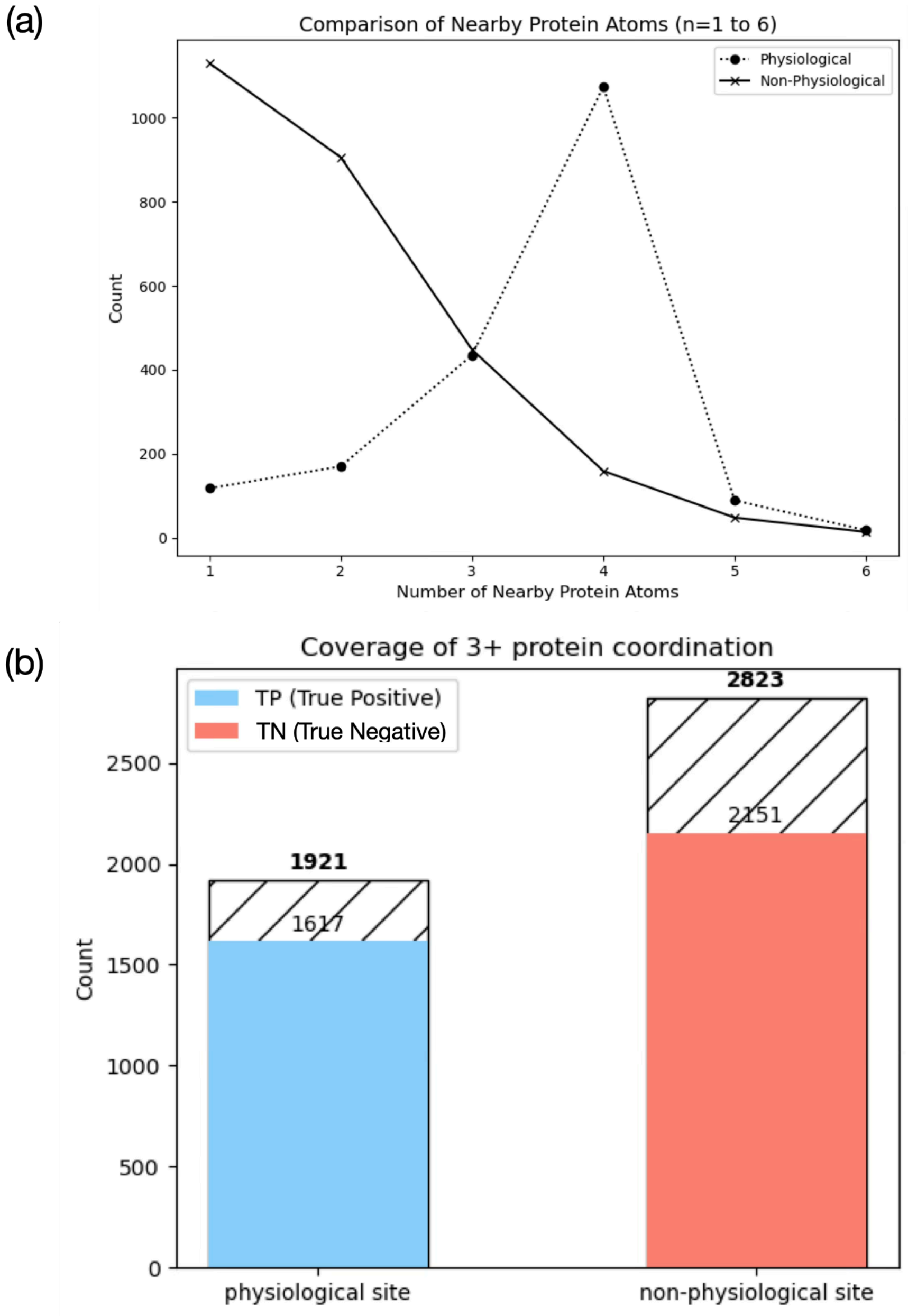

Figure S22. Rationale and validation of the ≥3 protein-coordination threshold for identifying biological metal-binding sites. (a) Distribution of nearby coordinating protein atoms (N, O, S, Se) for annotated biological and non-biological metal-binding sites. Biological sites exhibit a pronounced enrichment for ≥3 coordinating atoms, whereas non-biological sites show the opposite trend. (b) Coverage achieved using the ≥3-coordination threshold, validated on an independent annotated dataset. The threshold correctly identifies 1,697 of 1,921 biological sites and 2151 of 2,823 non-biological sites, supporting its effectiveness in filtering crystallographic artifacts and preserving stable physiological metal-binding patterns.

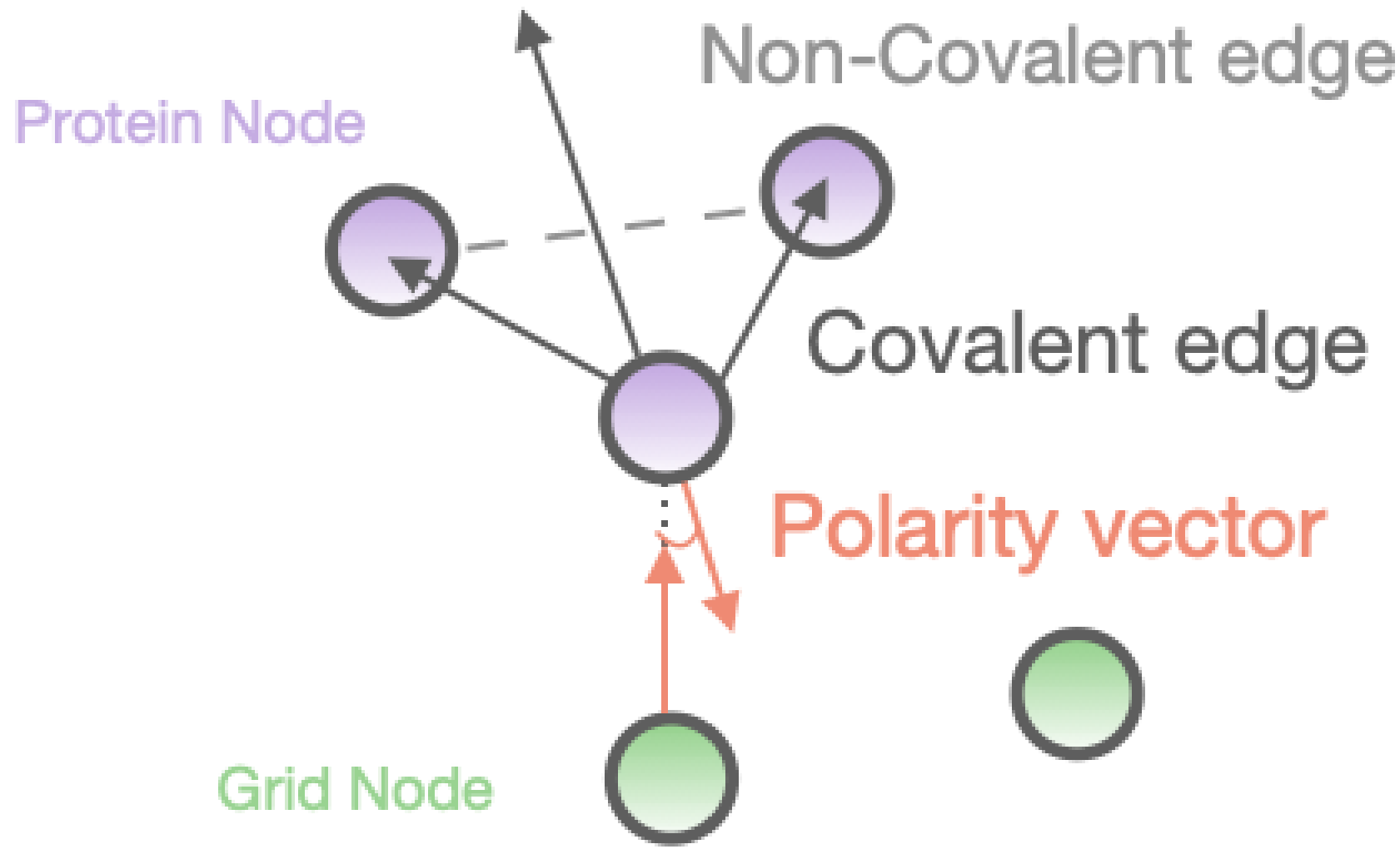

Figure S23. Illustration of the polarity-vector feature between protein and grid nodes. Protein nodes (purple) are connected by covalent (solid) and non-covalent (dashed) edges. For each protein node, its polarity vector is defined as the opposite of the sum of the unit vectors along its covalent bonds, while for each grid node (green) it is defined as the unit vector pointing toward the corresponding protein node. For protein–protein and protein–grid edges, we encode the angle between their polarity vectors via $(\sin\theta,\cos\theta)$; for edges between two grid nodes, this feature is set to (0,0).

Tabel S1. Summary of datasets and benchmark sets used in this study

Set name	Source	Selection / definition	Main purpose	Notes
**Training set**	Biologically filtered dataset	Entries released up to 2018-12-31, after 30% sequence clustering; single-chain only	Train the RF pruning model and EGNN optimization model	16,196 entries; only single-chain structures were used due to computational constraints
**Validation set**	Biologically filtered dataset	Entries released from 2019-01-01 to 2021-03-30; clusters overlapping with training removed; multi-chain allowed	Hyperparameter selection and model tuning	Strictly non-overlapping with training in sequence space
**Test set (= General PDB test set)**	Biologically filtered dataset	Entries released from 2021-03-31 onward; clusters overlapping with training/validation removed; multi-chain allowed	Held-out evaluation	Strict temporal and sequence separation from training data
**X-ray subset**	General PDB test set	Test entries solved by X-ray crystallography	Robustness analysis by experimental modality	Represents cleaner local geometry conditions
**Cryo-EM subset**	General PDB test set	Test entries solved by cryo-EM	Robustness analysis by experimental modality	Used to test tolerance to coordinate noise / medium-resolutio n uncertainty
**Model structure benchmark**	Targets derived from the General PDB test set	Chain A only; direct protein coordination only; AF3-modeled structures retained if global backbone RMSD ≤ 5 Å and local RMSD ≤ 3 Å within 5 Å of the metal site	Compare performance on experimental vs. AF3-predicted structures	Used for AF3 robustness analyses
**AF3 prompted metal-place ment benchmark: Negative set**	Subset of Boltz-1 test set	318 proteins with no annotated bound metal ions; AF3 prompted with one Zn	Test whether AF3 spuriously places metals in non-binding proteins	Used for non-binding discrimination
**AF3 prompted metal-place ment benchmark: Positive set**	Subset of Boltz-1 test set	Proteins containing a single metal-binding site; not restricted to single-chain proteins; multiple protein chains, DNA, or RNA allowed; homo-oligomer cases allowed with one representative chain-site instance selected	Test whether AF3 predicts the correct site and metal type	Final set size: 82 cases (Ca 19, Mg 21, Zn 22, Fe 6, K 4, Mn 7, Cu 3)

Table S2. Coordinate accuracy of predicted metal-binding site coordinates at method-recommended probability thresholds

Method	Cutoff (Å)	Matched (Total = 2,938)	Median(Å)	IQR (Å)	Q1–Q3 (Å)	MAD (mean ± std) (Å)
PrOMet	2.0	2,340	0.620	0.520	0.415–0.935	0.726 ± 0.427
Metal3D	2.0	2,017	0.484	0.617	0.263–0.879	0.625 ± 0.460
BioMetAll	2.0	384	1.412	0.783	0.912–1.694	1.313 ± 0.481
PrOMet	5.0	2,444	0.648	0.586	0.427–1.013	0.835 ± 0.647
Metal3D	5.0	2,072	0.502	0.713	0.265–0.978	0.753 ± 0.732
BioMetAll	5.0	851	2.757	1.623	1.953–3.575	2.745 ± 1.172

1) Thresholds were applied as: $p = 0.5$ for PrOMet, t = 0.5 for Metal3D, c = 0.5.

Table S3. Comparison of pruning-stage models across decision thresholds.
Pruning performance of Random Forest, LightGBM, and MLP models is summarized using three metrics: metal-site coverage, retained grid ratio, and false/true ratio. Metal-site coverage denotes the fraction of annotated metal-binding sites retained after pruning, where a site is counted as retained if at least one grid point remains within 2 Å of the annotated metal position. Retained grid ratio indicates the proportion of sampled grid points remaining after filtering relative to the total number of grid points. False/true ratio represents the number of retained negative grid points divided by the number of retained positive grid points. Random Forest and LightGBM maintain very high metal-site coverage across thresholds while progressively reducing the retained grid set, whereas MLP yields much lower false/true ratios only by substantially sacrificing metal-site coverage.

Model	Threshold	Metal-site coverage(↑)	Retained grid ratio(↓)	False/True grid ratio
RandomForest	0.1	0.993	0.164	208.675
RandomForest	0.2	0.993	0.088	114.601
RandomForest	0.3	0.991	0.057	74.985
RandomForest	0.4	0.990	0.039	52.185
RandomForest	**0.5**	0.988	0.028	37.018
RandomForest	0.6	0.986	0.019	26.049
RandomForest	0.7	0.983	0.013	17.717
RandomForest	0.8	0.978	0.008	11.223
RandomForest	0.9	0.959	0.004	5.892
LightGBM	0.1	0.993	0.083	107.551
LightGBM	0.2	0.991	0.054	70.878
LightGBM	0.3	0.990	0.040	53.345
LightGBM	0.4	0.989	0.032	42.138
LightGBM	**0.5**	0.987	0.025	33.870
LightGBM	0.6	0.986	0.020	27.033
LightGBM	0.7	0.984	0.015	20.978
LightGBM	0.8	0.981	0.011	15.302
LightGBM	0.9	0.971	0.007	9.195
MLP	0.1	0.929	0.002	2.713
MLP	0.2	0.906	0.001	1.550
MLP	0.3	0.891	0.001	1.192

MLP	0.4	0.874	0.001	0.910
MLP	**0.5**	0.845	0.001	0.615
MLP	0.6	0.809	0.000	0.427
MLP	0.7	0.751	0.000	0.302
MLP	0.8	0.675	0.000	0.207
MLP	0.9	0.543	0.000	0.135

Table S4. Overview of dataset composition after chronological splitting and cluster-based redundancy filtering. Training data were restricted to single-chain structures due to memory constraints, and entries containing more than 50,000 atoms were removed from all splits to ensure tractable data loading and preprocessing. Chronological cutoffs (2019-01-01 for training/validation and 2021-03-31 for validation/test) ensured strict temporal separation, while sequence-cluster filtering prevented redundancy between training and evaluation datasets.

Set	Chronological Range	No. of PDB Entries	No. of Chains	Notes
**Training**	– 2018-12-31	16196	Sinlge chain PDB	Only single-chain structures used due to memory constraints
**Validation**	2019-01-01 – 2021-03-30	926	Multi-chain allowed	Training-overlap clusters(seq id. 30%) removed
**Test**	2021-03-31 – Present	968	Multi-chain allowed	Strictly non-overlapping(seq id. 30%) with train/val clusters

Table S5. Distribution of metal-binding sites across training, validation, and test sets after applying temporal splitting, cluster-based redundancy removal, and coordination-based filtering (≥3 coordinating protein atoms). Counts are reported for each metal ion as well as total sites in each dataset split.

Metal Ion	Training	Validation	Test	Total
Zn	7557	1081	1579	10,217
Ca	7667	727	735	9,129
Mg	3434	634	550	4,618
Mn	1651	129	160	1,940
Cu	1032	101	30	1,163
Fe	707	194	90	991
Co	452	58	5	515
Ni	36	9	4	49
K	29	20	0	49
**Total**	22,565	2,953	3,153	28,671

Table S6. Features for Randomforest training. For each grid, the following features were included as input variables.

ID	*Feature group*	*Feature name*	*Distance thresholds (Å)*	*Dim.*	*Description*
1	Protein coordination	# of protein coordination atoms (N/O/S)	Cumulative counts within d, where d ∈ {2.5, 2.8, 3.0, 3.2, 3.5}	5	Number of protein N, O, and S atoms within distance d from the metal ion. (Ligand atoms excluded)
2	Ligand coordination	# of ligand coordination atoms (N/O/S/Se)	Cumulative counts within d, where d ∈ {2.5, 2.8, 3.0, 3.2, 3.5}	5	Number of ligand N, O, S, and Se atoms within distance d from the metal ion.
3	Ligand coordination	# of ligand negative ions	Cumulative counts within d, where d ∈ {2.5, 2.8, 3.0, 3.2, 3.5}	5	Number of negatively charged ligand atoms/ions within distance d from the metal ion.
4	Protein coordination	# of core-residue coordination atoms	Cumulative counts within d, where d ∈ {2.5, 2.8, 3.0, 3.2, 3.5}	5	Number of coordinating atoms from core residues (His, Cys, Asp, Glu) within distance d.
5	Protein coordination	# of backbone O coordination atoms	Cumulative counts within d, where d ∈ {2.5, 2.8, 3.0, 3.2, 3.5}	5	Number of backbone oxygen atoms that coordinate the metal within distance d.
6	Local distance geometry	Nearest nonpolar carbon distance	–	1	Distance from the metal ion to the closest nonpolar carbon atom.
7	Local distance geometry	Nearest backbone atom distance	–	1	Distance from the metal ion to the closest backbone atom (N, C, O, etc.).

8	Structural openness	Local RSA (relative solvent accessible area)	–	1	Relative solvent accessibility of residues surrounding the metal site.
9	Geometric patterns	# of core residues satisfying PA/PB/PAB criteria	–	1	Number of core residues (His, Cys, Asp, Glu) that satisfy predefined geometric constraints (PA, PB, PAB).
10	Second-shell environment	# of second-shell residues	3.2–5.0	1	Number of residues whose any atom lies within 3.2–5.0 Å from the metal ion.
11	Second-shell environment	# of second-shell core residues	3.2–5.0	1	Number of core residues (His, Cys, Asp, Glu) located in the second shell (3.2–5.0 Å).
12	Second-shell environment	# of second-shell backbone O coordination atoms	3.2–5.0	1	Number of backbone oxygen atoms coordinating the metal in the second shell (3.2–5.0 Å).

Table S7. Node and edge features for the GNN. In our GNN formulation, both protein atoms and spatial grids were treated as nodes. Edges were constructed by connecting each node to its 16 nearest neighbors based on Euclidean distance.

(a) Node Feature

Feature name	*Shape*	*Description*
**Atom positions**	(n_atoms, 3)	3D Cartesian coordinates of each atom.
**Amino-acid type**	(n_atoms, 30)	One-hot encoding of the amino-acid type of the residue to which the atom belongs (30 residue classes).
**Atom type**	(n_atoms, 120)	One-hot encoding of the atom type, including element, hybridization, and local chemical environment (120 atom classes).
**Sasa**	(n_atoms, 1)	Solvent-accessible surface area (SASA) of each atom.
**Secondary structure**	(n_atoms, 10)	One-hot encoding of the secondary-structure state of the residue (10 states).
**Atom chemical type**	(n_atoms, 65)	One-hot encoding of chemically defined atom categories (65 chemical types).
**Atom partial charge**	(n_atoms, 1)	Partial atomic charge assigned to each atom.

(b) Edge Feature

Feature name	*Shape*	*Description*
**Distance bin**	(n_edges, 150)	Binned inter-atomic distance between two nodes; distances in [0, 15] Å are discretized into 150 radial bins with width 0.1 Å.

**Edge type**	(n_edges, 4)	One-hot encoding of edge type (e.g., grid to grid, grid to protein, protein to grid, protein to protein)
**Covalent bond**	(n_edges, 1)	Binary indicator specifying whether the edge corresponds to a covalent bond.
**Angle between polarity vectors**	(n_edges, 2)	Two-dimensional encoding of the angle between the polarity vectors of the two nodes (e.g., sine/cosine of the angle).
